# Machine learning and metabolic modelling assisted implementation of a novel process analytical technology in cell and gene therapy manufacturing

**DOI:** 10.1038/s41598-023-27998-2

**Published:** 2023-01-16

**Authors:** Thomas Williams, Kevin Kalinka, Rui Sanches, Greg Blanchard-Emmerson, Samuel Watts, Lee Davies, Carol Knevelman, Laura McCloskey, Peter Jones, Kyriacos Mitrophanous, James Miskin, Duygu Dikicioglu

**Affiliations:** 1 Oxford Biomedica (UK) Limited, Windrush Court, Transport Way, Oxford, OX4 6LT UK; 2 Department of Biochemical Engineering, University College London, Bernard Katz Building, Gower Street, London, WC1E 6BT UK; 3 Department of Biosystems Science and Engineering, ETH Zurich, Mattenstrasse 26, 4058 Basel, Switzerland; 4 WattBE Innovations Limited, Winchester Business Centre, 10 Parchment Street, Winchester, SO23 8AT Hampshire UK

**Keywords:** Gene therapy, Biotechnology, Biochemical reaction networks, Machine learning

## Abstract

Process analytical technology (PAT) has demonstrated huge potential to enable the development of improved biopharmaceutical manufacturing processes by ensuring the reliable provision of quality products. However, the complexities associated with the manufacture of advanced therapy medicinal products have resulted in a slow adoption of PAT tools into industrial bioprocessing operations, particularly in the manufacture of cell and gene therapy products. Here we describe the applicability of a novel refractometry-based PAT system (Ranger system), which was used to monitor the metabolic activity of HEK293T cell cultures during lentiviral vector (LVV) production processes in real time. The PAT system was able to rapidly identify a relationship between bioreactor pH and culture metabolic activity and this was used to devise a pH operating strategy that resulted in a 1.8-fold increase in metabolic activity compared to an unoptimised bioprocess in a minimal number of bioreactor experiments; this was achieved using both pre-programmed and autonomous pH control strategies. The increased metabolic activity of the cultures, achieved via the implementation of the PAT technology, was not associated with increased LVV production. We employed a metabolic modelling strategy to elucidate the relationship between these bioprocess level events and HEK293T cell metabolism. The modelling showed that culturing of HEK293T cells in a low pH (pH 6.40) environment directly impacted the intracellular maintenance of pH and the intracellular availability of oxygen. We provide evidence that the elevated metabolic activity was a response to cope with the stress associated with low pH to maintain the favourable intracellular conditions, rather than being indicative of a superior active state of the HEK293T cell culture resulting in enhanced LVV production. Forecasting strategies were used to construct data models which identified that the novel PAT system not only had a direct relationship with process pH but also with oxygen availability; the interaction and interdependencies between these two parameters had a direct effect on the responses observed at the bioprocess level. We present data which indicate that process control and intervention using this novel refractometry-based PAT system has the potential to facilitate the fine tuning and rapid optimisation of the production environment and enable adaptive process control for enhanced process performance and robustness.

## Introduction

Process analytical technology (PAT) is a concept that covers a range of tools and techniques that facilitate the improved design, analysis and control of pharmaceutical manufacturing processes through the monitoring of critical quality and performance attributes^1^. Different technological approaches are increasingly being investigated and adopted for PAT in the pharmaceutical and biotechnology industries to enable enhanced process control and understanding, the implementation of quality by design, the promotion of real time release and continuous manufacture^2^. PAT strategies that enable real-time monitoring and feedback control of processes facilitate a more flexible and adaptable approach to manufacturing, which in turn makes real-time in-process PAT particularly applicable in processes where there is a need for the process control strategy to cope with numerous interacting variables to maximise process robustness, such as for the manufacture of complex biological products, like viral vectors, or autologous cell therapies where significant donor-to-donor variability is frequently encountered^3^. The principal objective of PAT is to efficiently monitor and control critical process parameters (CPPs), affecting critical quality attributes (CQAs), to consistently ensure final product quality. PAT comprise a critical part of a broader initiative of U.S. Food and Drug Administration (FDA) which outlines how pharmaceuticals should be manufactured in the twenty-first century in light of current good manufacture practices (cGMPs); the initiative seeks to implement a contemporary, risk-based regulatory framework for the oversight of pharmaceutical manufacturing^4^. The often dynamic and complex nature of the products produced in the biopharmaceutical industry means that the application of PAT solutions have been more complex and challenging and, consequently slower to be implemented in real-life settings compared to other industries, despite there being a high demand for them to assist the streamlining of biopharmaceutical manufacturing processes^5^.

There are an increasing number of examples in the pharmaceutical industry and, broadly, in the biotechnology sector demonstrating the successful deployment of PAT for improved bioprocessing. Pais et al. applied fluorescence spectroscopy to monitor adeno-associated virus production in the insect cell-baculovirus expression vector system^6^, Webster et al. developed models that predict concentrations of key metabolites in Chinese hamster ovary (CHO) cultures using Raman spectroscopy^7^ and Mercier et al. evaluated the use of dielectric and near-infrared spectroscopy to predict cell and metabolite concentrations in perfusion cultures^8^. These applications are examples of so-called ‘soft sensors’, which combine software models that infer information about process variables, which cannot be measured directly in an online fashion, but rather are based on an understanding of the relationship of that variable with other variables which can be measured by available hardware sensors^9^. In the case of spectrometry, there is a relationship between the measured spectra and certain media components, which consequently can support inference models where information can be correlated with key process performance attributes such as cell viability and product concentration^6^. This key inference step is usually realised using data-driven models obtained from multivariate data analysis (MVDA). The wider adoption of soft sensors into biopharmaceutical manufacturing processes would be expected to not only enable improved real time monitoring but also potentiate the broader implementation of dynamic process control, which could be increasingly automated following the vision of the PAT principles. The potential for this was demonstrated by Liu et al., who developed a multivariate statistical process control model based on Raman spectroscopy to predict batch variability in IgG antibody production processes with CHO cells^10^ that were capable of detecting microbial contaminations in a batch early, which would normally only be diagnosed after 200 h by traditional methods. Although spectrometric methods represent the majority of soft sensing applications that have been reported to date, there is also a growing interest in evaluating the performance of refractometric devices as analytical modalities due to their relatively low cost to implement, the simplicity and ease of instrument set-up and operation and their ability to enable highly accurate and rapid measurements, potentiating their use for adaptive process control^11–13^.

While such data-driven models that are developed using MVDA and machine learning are subject of a lot of research, there is a second approach of computer modelling which has been studied for advanced understanding and control of mammalian cell cultures, namely (semi) mechanistic modelling, involving the use of metabolic fluxes and reaction kinetics^9^. Such models can provide information about the metabolic state of cells and assess, for instance, what media compounds to use for bioprocess control and the consequences for cell growth and product biosynthesis. Teixeira et al. applied a semi mechanistic model integrating detailed information about the metabolism of baby hamster kidney (BHK) cells to exert bioreactor control in cultures for recombinant protein production^14^. Complex, purely mechanistic, metabolic flux networks and even genome scale metabolic networks of mammalian cells have since been developed^15,16^. However, due to a lack of integration of extracellular stimuli their direct application for mammalian bioprocess control has rarely been described.

**Figure 1:**
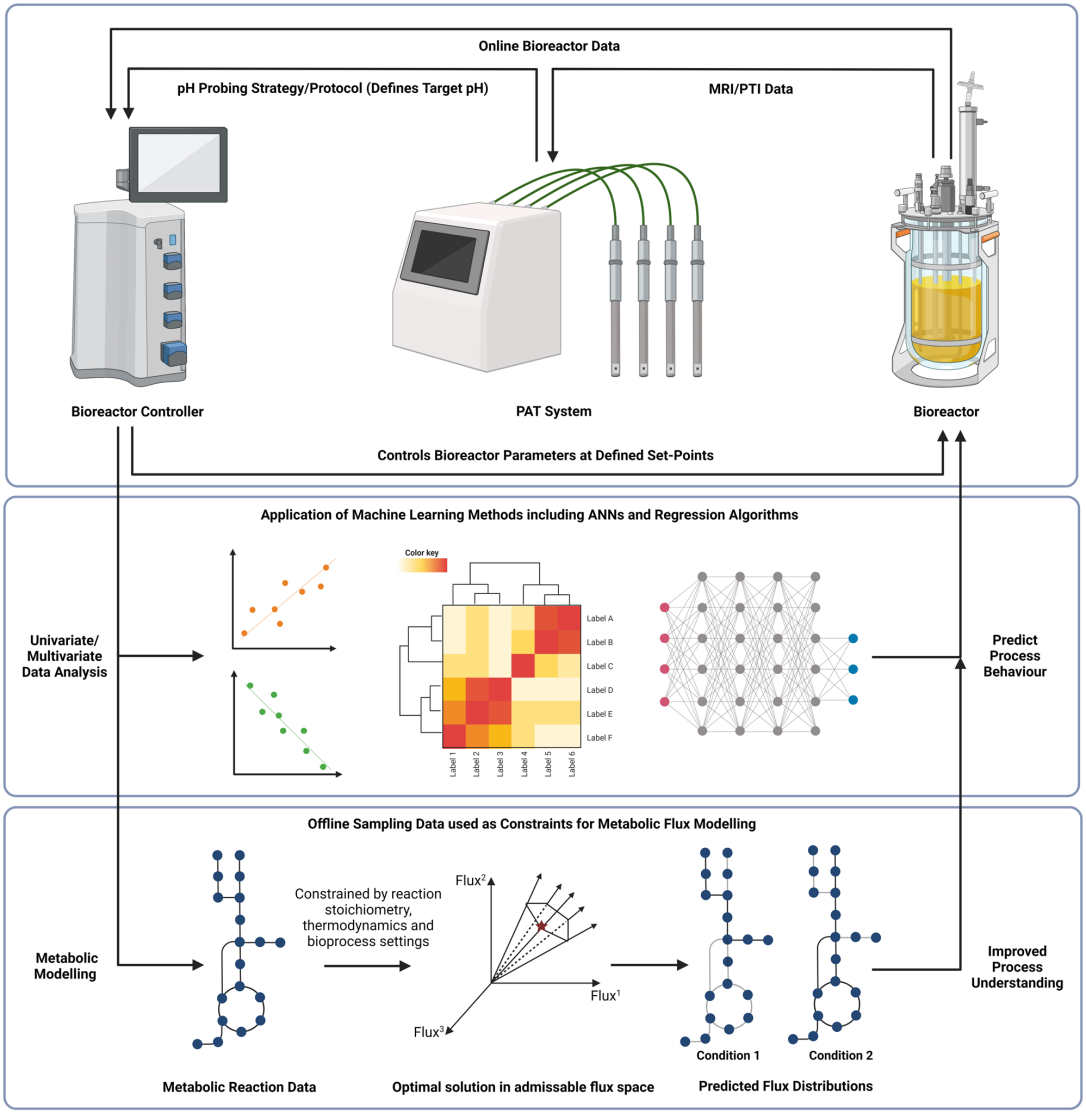
Schematic of the integration of the Ranger RI system with a bioreactor and a bioreactor controller unit. The PAT has the ability to adapt bioprocessing conditions based on the PTI/MRI data collected during an ongoing bioprocess. Figure made using BioRender.

The Ranger Refractive Index (RI) PAT system (WattBE Innovations, Hampshire, UK) utilised in this work is a novel advanced RI profiling technology which combines the measurement of the refractive index of a cell culture, provided via an in situ optical probe, with a process parameter probing strategy to provide a tool that facilitates the assessment and control of cell culture activity^17^. The system is able to detect changes in cell culture composition related to cellular metabolism and compare cellular activity under different bioreactor operating conditions, enabling a deeper understanding of the real-time culture requirements for specific cell lines and products. Additionally, the Ranger RI system has the ability to integrate with bioprocesses equipment and adapt bioprocessing conditions to maximise the activity of the cell culture (Fig. 1). The signal that is detected by the Ranger RI system is termed the Process Trend Index (PTI). The PTI is essentially a function of the RI of the culture medium, which indicates the combined effect of all media components. The system works on the basis that increasing the rate of the PTI change that is being measured in the culture enables the cells to work at an increased rate, hypothesised to increase productivity. Filtering the change in PTI over time and fitting this to expected reaction kinetics provides a second value, termed the Metabolic Rate Index (MRI); a value that measures how PTI changes with time and, in effect, the rate at which the composition of the cell culture is changing. MRI is the derivative of a second order polynomial fitted to the raw sensor data. This is used to indicate the level of metabolic activity. The real-time monitoring of MRI enables the comparison of the activity of cellular processes between two time points (for further details see patent ref. PCT/EP2015/070025).

The Ranger RI system has previously been successfully deployed for high-yield protein production cell lines with long stationary production phases. In collaboration with the GSK Medicine Research Centre, it was demonstrated how the system could be used for automated closed loop feeding control in a CHO cell culture realising an on-demand feeding strategy in real time which met the culture’s requirements throughout the duration of the process^18^. Similarly, Biziato et al. used the system to apply an automated, adaptive feeding strategy in cultures of primary human T cells^19^, presenting the first proof of concept of this type of process control for autologous immunotherapy processing. In these settings, the Ranger RI system has controlled cellular activity through the management of the nutrient levels in the media following a short study of various bioreactor environmental parameters and has acted to increase the activity of cell cultures.

Here we describe the application of this novel RI profiling technology to in the context of Oxford Biomedica’s (OXB) proprietary suspension LentiVector platform with the objective of driving higher volumetric productivity and LVV titres through the increased control of manufacturing processes. A human embryonic kidney (HEK) 293 T-based producer cell line (PCL) was engineered to produce recombinant, vesicular stomatitis virus glycoprotein (VSV-G) pseudotyped, human immunodeficiency virus (HIV-1) based lentiviral vectors (LVV), containing a green fluorescence protein (GFP) encoding genome. A LVV production bioprocess was designed specifically for this work with parameters specifically selected to explore the full potential of the Ranger RI system for LVV applications. This is the first time this PAT system’s techniques have been used in real-time to control the environmental parameters of a cell culture with a production phase shorter than 5 days and the first time that the system has been used in the context of a viral vector production bioprocess. In this challenging setup, we looked to explore how the Ranger RI system would perform when there was no direct protein-related production with an obvious dominant impact on the PTI signal; instead, responses were only captured via general cellular activity. Here we apply a ‘parameter probing’ methodology, an early-stage concept developed to enable the tuning of operational conditions in real time to adapt processes for enhanced cellular activity, to determine whether improvements to the level of activity of an LVV PCL can be achieved. We combine empirical observations with advanced data analytics and semi-mechanistic modelling to determine whether this correlates with an increase in functional LVV titre and elucidate the underlying cellular mechanisms leading to the process level understanding of these observations.

## Material, methods and modelling methodologies

### Cell culture

A serum-free, suspension-adapted, HEK293T-based LVV PCL, used to produce recombinant, VSV-G pseudotyped, HIV-1-GFP based LVVs, was generated previously from an adherent HEK293T cell line, provided by Oxford Biomedica; all LVV encoding genetic elements were stably integrated into the host-cell genome. The PCL was cultured in FreeStyle 293 Expression Medium (Gibco, Thermo Fisher Scientific, Waltham, MA, USA) and cultivated in 5 L bioreactors (Applikon Biotechnology, The Netherlands). An Acridine Orange (total cell stain) and 4′,6-diamidino-2-phenylindole (DAPI, dead cell stain) staining procedure was used to determine viable cell concentration (VCC) and cell viability via analysis on an automated NucleoCounter NC200 (ChemoMetec, Denmark). Online bioreactor process parameters were recorded every minute using a supervisory control and data acquisition (SCADA) system. Offline samples were taken during daily monitoring activities and prior to key process steps to measure VCC, cell viability, cell aggregation, functional vector titre and the concentration of the p24 capsid protein. Additionally, concentrations of glucose, lactate, ammonium, sodium, potassium, glutamine, and glutamate denoted as [glucose], [lactate], [NH4+], [Na+], [K+], [Gln], [Glu], respectively, were measured using a Nova FLEX 2 (Nova Biomedical, Waltham, MA, USA).

### Lentiviral vector production

Recombinant, VSV-G pseudotyped, HIV-1-GFP based LVV production was induced in the LVV PCL via the addition of doxycycline to cell cultures in 5 L bioreactor. A secondary induction step, involving the addition of sodium butyrate to cell populations, was conducted to enhance transcription of the genetic elements encoding the LVV approximately 20 h following the addition of doxycycline. The HIV-1-based LVV containing supernatant was harvested approximately 44 h following the initial induction of LVV expression, clarified through a 0.45 µm filter and stored at − 80 °C for subsequent analysis.

### PAT measurements and protocols

PTI, derived from the RI of the cell culture, was calculated using in situ optical probes mounted within 5 L bioreactors. A data filtering algorithm was applied to the PTI measurements to automatically derive the MRI of the culture in real time. Measurements were taken every 5 s. The Ranger RI system was used to perform ‘parameter probing’, which was executed in two modes of operation, employing both pre-programmed and autonomous logic methodologies. The pre-programmed mode of operation followed a pre-defined script detailing a series of pH parameter changes scheduled against fixed process times, communicated to the bioreactor’s bio-controller unit (Applikon Biotechnology, The Netherlands) via an open platform communication (OPC) connection. The autonomous mode of operation involved the dynamic adjustment of parameter set points, in real-time, determined by the result observed following a previous parameter adjustment in the experiment, to hone into parameter settings which maximised the greatest level of cellular activity.

### Transduction and titration of LVVs

#### Functional LVV titre

Functional vector titres were determined via the transduction of adherent HEK293T cells, seeded in 12 well plates 24 h prior to transduction. Test samples were serially diluted in DMEM (Sigma-Aldrich, Merck, Burlington, MA, USA) supplemented with 8 µg/mL polybrene (Sigma-Aldrich, Merck, Burlington, MA, USA). Transduced cells were harvested 72 h following their exposure to the diluted viral vector preparations and analysed for GFP expression via flow cytometry using an Attune NxT acoustic focusing flow cytometer (Thermo Fisher Scientific, Waltham, MA, USA) using an optimised GFP detection protocol. Functional LVV particles have been expressed as the number of transducing units/mL, calculated using Eq. (1).

1 $$Titre \left( {TU/{\text{mL}}} \right) = \frac{{\left[ {\frac{\% Parent }{{100}} \times Conc. \ of \ cells \ prior \ to \ transduction \times Dilution factor} \right]}}{{Volume \ of \ vector \ added \left( {mL} \right)}}$$

#### Total LVV Particles

Quantification of the p24 capsid protein in LVV preparations was performed by an enzyme-linked immunosorbent assay (ELISA). p24 concentrations were measured with an Alliance HIV-1 p24 ELISA kit (Perkin Elmer, Waltham, MA, USA) and converted into total number of LVV particles using Eq. (2).

2 $$Total \,particles \left( {\frac{TP}{{{\text{mL}}}}} \right) = \left( {\frac{{p24 \,concentration\left( {\frac{{\text{g}}}{{{\text{mL}}}}} \right)}}{Molecular\, weight \,of\, p24}} \right) \times \left( {\frac{{Avogadro^{^{\prime}} s\, number}}{Number \,of \,p24 \,molecules \,per \,HIV \,particle}} \right)$$

*Molecular weight of p*24*:* 2.4 × 10^4^.

*Number of p*24 *molecules per HIV particle:* 2000.

### Metabolic modelling

#### Model

The HEK293 metabolic model developed by Martínez-Monge^20^, derived from the most recent human genome-scale metabolic reconstruction Recon 2.2^16^, was used. This model represented 354 reactions between 335 metabolites and was extended in this work to include two additional exchange reactions for sodium and potassium ions, respectively. This modification had an impact on the flux through 23 reactions (Supplementary Table 1).

#### Analyses

Flux balance analysis (FBA)^21,22^ was performed using the COBRA toolbox (v. 3.0) running under MATLAB (v. 9.8) , using the Gurobi optimiser (v. 9.1.1) as the mathematical solver. The objective function to be maximised was the growth rate flux. The upper and lower bounds of exchange reactions, for which experimental data were available, were constrained accordingly. The constraints for the upper bounds of several exchange reactions that allow the uptake of nutrients, for which empirical data was not available, were determined using the flux variability analysis (FVA). Amino acid exchange fluxes were among these critical constraints. FVA^23^ was performed using the same platform. The flux ranges of the amino acid exchange reactions provided by the FVA solution were evaluated based on typical amino acid concentrations in cell culture media; a few hundred milligrams per litre up to slightly more than 1 g/L were accepted to be the typical range. When the FVA solution range exceeded this, the amino acid exchange fluxes were constrained with tighter bounds and the FVA was repeated. FVAs led to the range of $\pm$ 1 mmol/(g CDW × h) for all amino acids except for valine and isoleucine, which were constrained to the range of between $\pm$ 0.5 mmol/(g CDW × h).

#### Identifying feasible ranges for exchange fluxes of medium components of undefined composition

Genome scale metabolic models are highly underdetermined systems. Consequently, the predictions we make on the metabolic state of the cells often represent a range of available solutions that satisfy certain criteria equally well while achieving an optimum goal, which, in this case, would be optimum cellular growth rate. From a practical point of view, the more of these criteria we can identify and constrain to meet specific requirements, the narrower this range of possible solutions will get, thus allowing us to improve the precision of our understanding of the metabolic behaviour of the cell at any given metabolic snapshot. However, in a bioprocess setting, information, and data on many such criteria essential for metabolic modelling could be unavailable. There are different reasons for the absence of such information and one very prominent cause is the proprietary nature of the process settings and operations. Knowledge of the specific chemical composition of cell culture medium harbours valuable insight into how cellular metabolism behaves, and thus is critical in constraining metabolic models to achieve good predictive capability. However, the specific compositions of such mediums are often proprietary and not disclosed, complicating this process. Due to this, the constraints of critical model inputs, for which no experimental data was available, were estimated using FVA in this analysis. The amino acid fluxes were found to be particularly critical, and, therefore, the focus was set on these.

Since the only external input factor that was varied across the experiments was the cell culture pH profile, a strategy was needed to incorporate extracellular pH information into the metabolic model (Fig. 5b). This strategy allowed us to implement, for the first time, a strategy which accounted for the metabolic differences instigated by the extracellular pH at which the cell culture was maintained. Furthermore, amino acid requirements as determined by the respective ranges of the uptake fluxes were strongly associated with the incorporation of the sodium and potassium exchange reactions in the model.

#### Introducing pH as a constraint into the metabolic model

The following methodology was proposed to address the challenge: every cell exports a fixed number of protons, which in turn contributes to the measurement of extracellular pH. This constant and continuous proton export leads to an increase in the extracellular proton concentration which, if not counteracted, would cause a pH decrease in the extracellular environment. Since the quasi-steady state working assumption must hold, there needs to be an alternative route to counteract (or neutralise) this proton accumulation and maintain pH at the fixed control setpoint. A bicarbonate buffering system was used in the bioprocesses, therefore HCO_3_^−^ was selected as the neutralising compound, which needed to be exported, resulting in a steady equilibrium, i.e., a defined pH value. At a constant proton efflux, the bicarbonate efflux was specifically tuned to achieve the target pH value. Namely, changes were introduced to the constraints of two exchange reactions, those for H^+^ and HCO_3_^−^. First, the true pH value of the cell culture at the time point of interest was converted into the corresponding molar proton concentration (A). The proton export flux, which was set as a fixed constraint for the sake of comparability, was converted into a molar concentration (B) as well, and the difference between concentrations (A) and (B) was calculated. As (B) was consistently larger than (A), it was assumed that a certain amount of organic base must be exported to neutralise the excessive extracellular protons and maintain the pH. Based on the information that a bicarbonate buffering system was used in the bioprocesses, HCO_3_^−^ was selected as the neutralising compound. Under the assumption that 1 mol of base neutralises 1 mol of acid, the molar concentration of HCO_3_^−^ equivalent to the absolute difference between (A) and (B) was computed and subsequently converted into a flux value as described elsewhere. This flux was set as fixed constraint in FBA to simulate HCO_3_^−^ export.

Prior to its utilisation in this work, a proof-of-concept study was conducted where two FBAs were run to represent the same experimental setup, which only differed in the HCO_3_^−^ efflux constraint, representing different pH values of 6 and 7. The distribution of the fluxes for 26 reactions varied between the two FBA solutions (Supplementary Table 2). In all flux comparisons, absolute differences in fluxes between any two conditions were computed. As preliminary analysis, flux distributions obtained from FBA for different conditions were evaluated in a 2 by 2 factorial design: in the presence or absence of pH taken into account by the model, and Na^+/^K^+^ exchange reactions accounted for in the model or not (Supplementary Tables 3, 5, 7, 9).

#### Visualisation of metabolic pathway fluxes

Visualisation maps were generated in Escher-FBA^24^. The flux values of all reactions in the FBA solution were set as constraints for a new MATLAB model, which was then converted into a JSON model file using COBRApy (v. 0.21.0). The JSON model was uploaded into Escher-FBA and laid onto the RECON1 Escher map accessed from the BiGG Models database.

### Bioprocess data analysis

*Data and platforms.* The data analysis was conducted using Python (v. 3.8.7), R (v. 4.0.3) and RStudio (v. 1.1.463). Other supporting Python packages employed were “gplearn” (v. 0.4.1), “Lazy Predict” (v. 0.2.9), “NumPy” (v. 1.19.1), “pandas” (v. 1.2.3), “seaborn” (v. 0.11.1), “SHAP” (v. 0.38.1), “scikit-learn” (v. 0.23.1), “cowplot” (v. 1.1.0), “desiderata” (v. 0.38.0), “dplyr” (v. 1.0.2), “ggplot” (v. 3.3.2), “reshape2” (v. 1.4.4), “stringr” (v. 1.4.0).

A total of 22 upstream experiments were performed (bioprocess duration range of 66.15–69.25 h); 18 of the experiments were conducted as part of the process development phase and four experiments executed as part of the subsequent validation phase. Essentially, the process development phase involved the completion of experiments to develop a process able to enhance the metabolic activity of the cell culture compared to the ‘unoptimised’ process. The validation phase involved a direct comparison of the process that had been optimised for high levels of cellular activity to the ‘unoptimised’ process. For every bioprocess run, three subsets of data were acquired: bioreactor SCADA data, Ranger RI system data and offline sampling data. The bioreactor SCADA data comprised once per minute online records of elapsed process time (EPT), cumulative alkali addition, cumulative air addition, cumulative CO_2_ addition, cumulative N_2_ addition, cumulative O_2_ addition, measured dissolved oxygen, measured pH, measured stirrer speed, measured temperature, air flow rate, CO_2_ flow rate, N_2_ flow rate, O_2_ flow rate, dissolved oxygen set point, pH set point, stirrer speed set point and temperature set point. Three parameters were extracted from the Ranger RI system and data were recorded every five seconds: EPT, PTI and MRI. The offline sampling data collected for the development experiments were records of VCC, cell viability and cell aggregation at five time points of each bioprocess. For the four validation experiments records were collected at seven time points. In addition to VCC, cell viability and cell aggregation, there were records of vector functional titre, concentration of the p24 capsid protein and concentrations of glucose, lactate, ammonium, sodium, potassium, glutamine, and glutamate denoted as [glucose], [lactate], [NH4+], [Na+], [K+], [Gln], [Glu], respectively.

In the forecasting and regression experiments either MRI or PTI is used as the output variable; the cumulative alkali addition, cumulative air addition, cumulative CO_2_ addition, cumulative N_2_ addition, cumulative O_2_ addition, measured dissolved oxygen, measured pH, measured stirrer speed, measured temperature, air flow rate, CO_2_ flow rate, N_2_ flow rate, and O_2_ flow rate were used as the input variables as necessary.

### Online bioprocess data pre-processing

#### Handling of missing data

For the SCADA datafiles, the low rate of missingness allowed flexibility in the method of choice and simple moving average was selected as imputation method. The fractions of missing data in the MRI data were larger than that observed in the SCADA datafiles on average since the Ranger RI system is programmed to not record MRI data generated immediately following a modification to a parameter setting. The percentage of missing data, resulting from pH set point changes, ranged from 0% up to 53% across the various experiments, related to the number of pH changes that were made during the processes. MRI datafiles with extensive missingness (defined as more than 15% of data missing) were excluded from further analysis in order to avoid imputation-related information gain or loss in data analysis downstream of this task.

Univariate time series imputation was conducted using “imputeTS” (v. 3.1)^25^. For missing data at only a single time point, imputation was done by simple moving average. The sections of missing data in the MRI measurements were imputed by Kalman smoothing on a state space representation using “auto.arima” in R “forecast” (v. 8.3). Details on this are shown in Supplementary Figs. 1 and 2.

#### Data condensation

In order for the timeframes of the time series data to match in length and in time step size, the Ranger RI system dataset, where data were recorded every five seconds, was condensed to a data frequency of once every minute by computing the mean of the respective variable for every minute and was then merged with the remaining dataset. This merged dataset of each bioprocess run will henceforth be referred to as online data.

#### Feature selection

Different features may or may not bring in different information regarding a bioprocess run depending of the experiment that was conducted, and the identification of informative process parameters, i.e. features and running a sanity check on them prior to their utilisation in data analytics and modelling can become a non-trivial task^26^. In order to address this, a preliminary feature selection step was included in the process. sp_dO, sp_Stirrer and sp_Temperature were all removed prior to the analysis since these parameters were kept constant across all runs, hence would not carry any information. Dynamic time warping^27,28^ (“dtw” v. 1.22–1.23 in R) and Pearson correlation were used to detect multicollinearity among the online parameters (features) and investigate the similarity to/correlation with the target variables MRI/PTI. Pearson correlation was used to compare and contrast the results obtained by dynamic time warping in order to demonstrate the role of time in the identification of multicollinearity in the dataset. Each bioprocess was investigated individually for correlations within itself, and correlations were also sought across all available datasets. Correlation analysis led to the removal of EPT and sp_pH from the datasets due to high correlation with other features kept in the dataset. m_Stirrer and m_Temperature were excluded, too, since these features never showed significant temporal variation.

### Online data processing

#### Multiple forecasting

The artificial recurrent neural network architecture long short-term memory (LSTM) was applied and implemented using “Keras” (v. 2.4.3) in Python. Datasets of each process run were converted into a set of time windows; this was done separately for the features and the target variable to enable different window sizes for training and forecasting. Forecasting accuracy was evaluated using the root mean square error (RMSE) between forecasted and true data points. Two training approaches were used; one providing a training set of subsequent windows that differ by only one time step (“sliding window”). another one obtained by shuffling the windows of a training set. The randomised model training substantially decreased the loss during training and led to a much more steady and faster converging learning curve (Supplementary Fig. 3). The improved training performance in turn also improved forecasting precision, given by RMSE, by up to one order of magnitude since the entire process was used for learning while the sliding window approach only allowed the model to learn from an early given fraction of the process, having the information in the remaining time points to be never learned by the model, thus resulting in poor forecasting performance. The LSTM models were cross-validated using “scikit-learn” (v. 0.23.1) package employing stacked cross-validation.

#### Regression

Regression analyses were conducted with the “XGBoost” package (v. 1.1.1) in Python^29^. The coefficient of determination (R^2^) was used as an estimate of how well the model predictions approximate the underlying training data. RMSE was applied to evaluate model predictions for the target variable based on feature values it had not seen for training. The decision for the selection of the regression method to use was facilitated by Lazy Predict for Python. XGBoost Regressor was selected among regression methods with similar performance based on the metrics R^2^, RMSE and running time generated by the “LazyRegressor” function on a few randomly chosen process runs (Supplementary Fig. 4).

#### Univariate forecasting

Univariate forecasting was performed using the “pmdarima”^30^ (v.1.8.0) and “sktime” (v. 0.5.3) packages^31^ in Python. For each forecasting task one hour of past data was used for training to make a forecast one hour into the future. Forecasting accuracy was evaluated using the RMSE between forecasted and true data points.

### Statistical analysis

Statistical analysis was performed using Prism v9.1 (GraphPad Software, San Diego, CA, USA). Graphs depict the mean ± one standard deviation. Differences between two means were evaluated using unpaired two-tailed Student’s t-tests. Results were considered statistically significant when *p* values were less than 0.05 (*), 0.01 (**), 0.001 (***), 0.0001 (****).

## Results and Discussion

### Relationship between cell culture pH and metabolic activity

**Figure 2:**
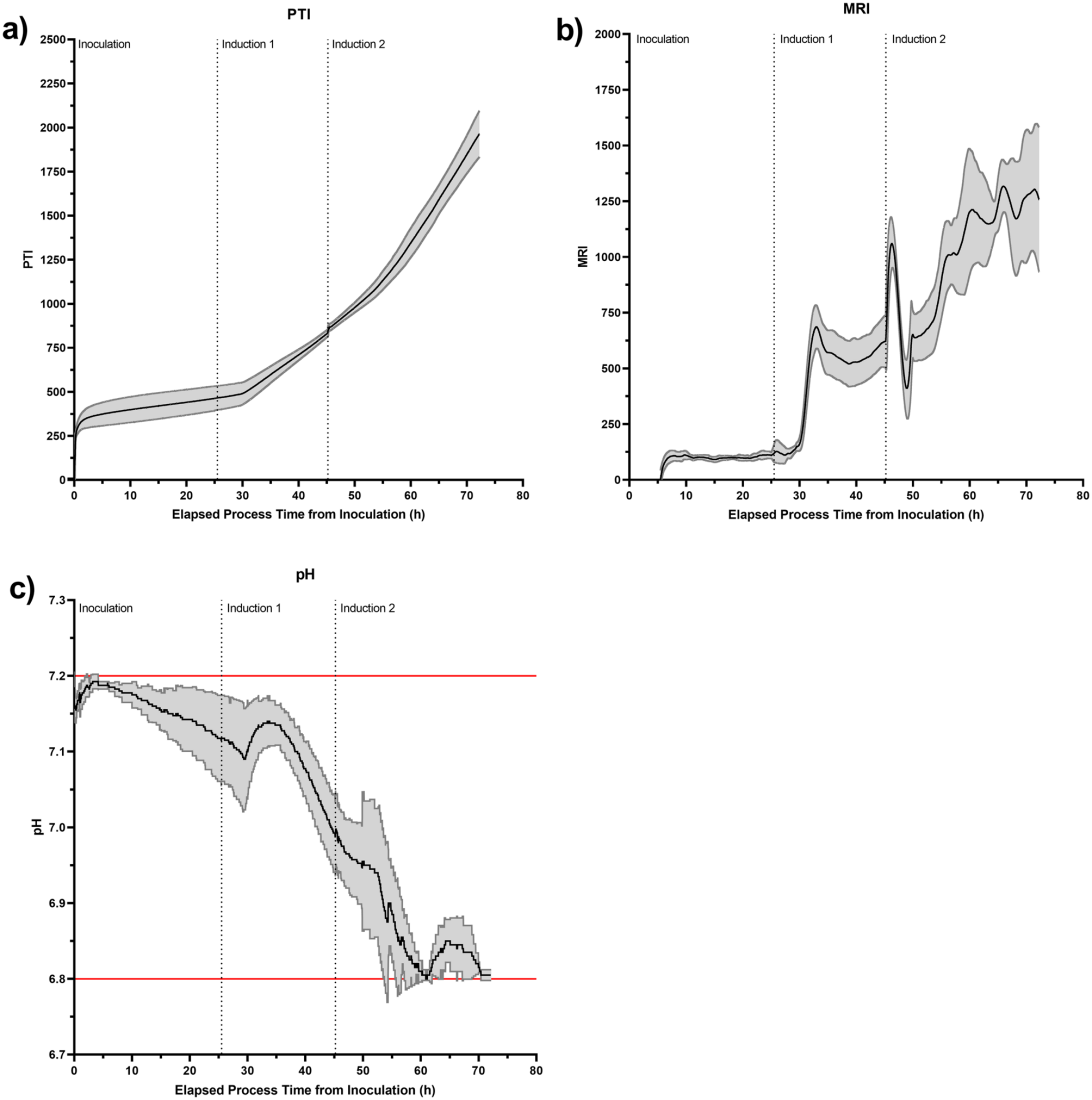
Unoptimised HEK293T-based PCL HIV-1-GFP LVV production bioprocess involving the passive monitoring of (**a**) PTI (**b**) MRI and (**c**) pH to gain baseline monitoring data. Black traces indicate the mean of four replicate bioreactor processes and grey traces indicate ± 1 standard deviation of the mean. Horizontal red lines indicate pH dead-band. Vertical dashed black lines indicate key process events.

A preliminary analysis was conducted to identify candidate process parameters that may be responsible for the variations in the metabolic activity monitored by the Ranger RI system. PTI and MRI data were collected in real-time from the HEK293T-based PCL HIV-1-GFP LVV production bioprocess to provide baseline monitoring information of the cellular activity. Strong PTI and MRI signals were obtained and similar profiles were observed in all production runs, indicating equivalent cellular activity between replicate processes (Fig. 2a and b); an average coefficient of variation of 9.9% was determined between the four replicate processes for PTI. PTI and MRI profiles represented three distinct phases of activity: (1) 0–30 h, (2) 30–45 h and (3) 45 h—harvest separated by two induction steps: (1) transcriptional activation of the genetic elements encoding the HIV-1-GFP LVV and (2) transcriptional enhancement of these genetic elements, indicating that the Ranger RI system was detecting genuine changes in the media composition reflective of the cellular process. The rapid oscillation in MRI observed immediately following ‘Induction Phase 2’ is attributable to a large volumetric addition of sodium butyrate at this timepoint, resulting in a rapid change in the refractive index of the culture, and hence PTI and MRI, and is likely not indicative of true metabolic changes during this short period. A wide pH dead-band of 7.00 ± 0.20 was employed for the duration of the process and in all runs the bioreactor pH declined from an initial post-inoculation pH of approximately 7.20–6.80 during the LVV production phase (Fig. 2c). Whilst a causal relationship could not be ascertained at this point, a decrease in culture pH was accompanied by an increase in metabolic activity indicated by the PTI and MRI signals. Bioreactor pH, therefore, was identified as a suitable process parameter for investigation in this LVV production process. It should be acknowledged that other monitored online parameters, such as air and oxygen gas flow rates, also correlated with an increase in PTI and MRI during these initial baseline monitoring experiments. However, pH was selected as the key parameter of interest to test the Ranger RI systems ability to adapt bioprocessing conditions to maximise the activity of the cell culture, since the utilisation of lower pH set-points (pH 6.00–6.80) has been recently reported to increase LVV production compared to neutral conditions^32–34^.

### Evaluation of pre-programmed and autonomous, real time, pH control strategies to maximise cell culture activity

**Figure 3:**
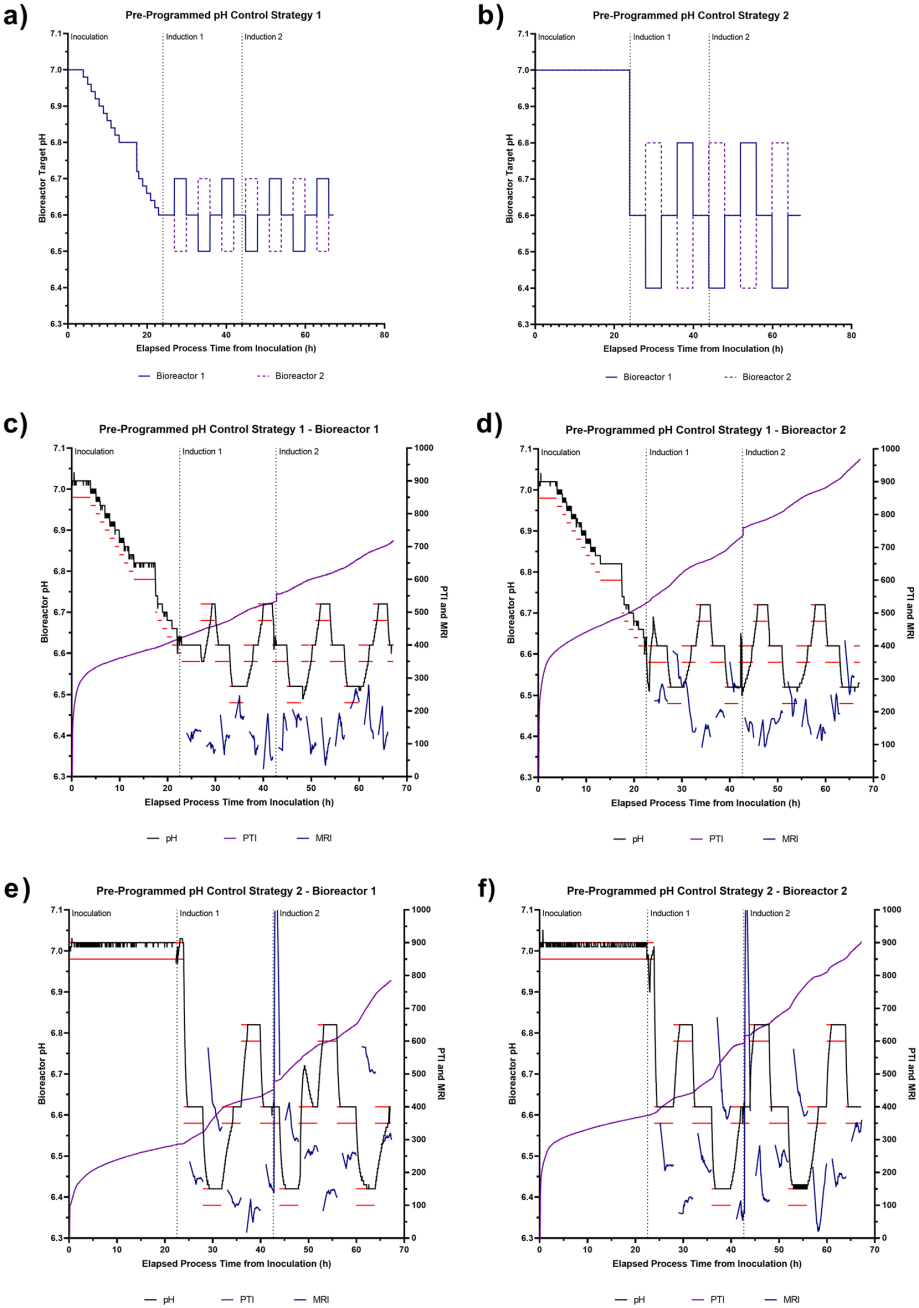

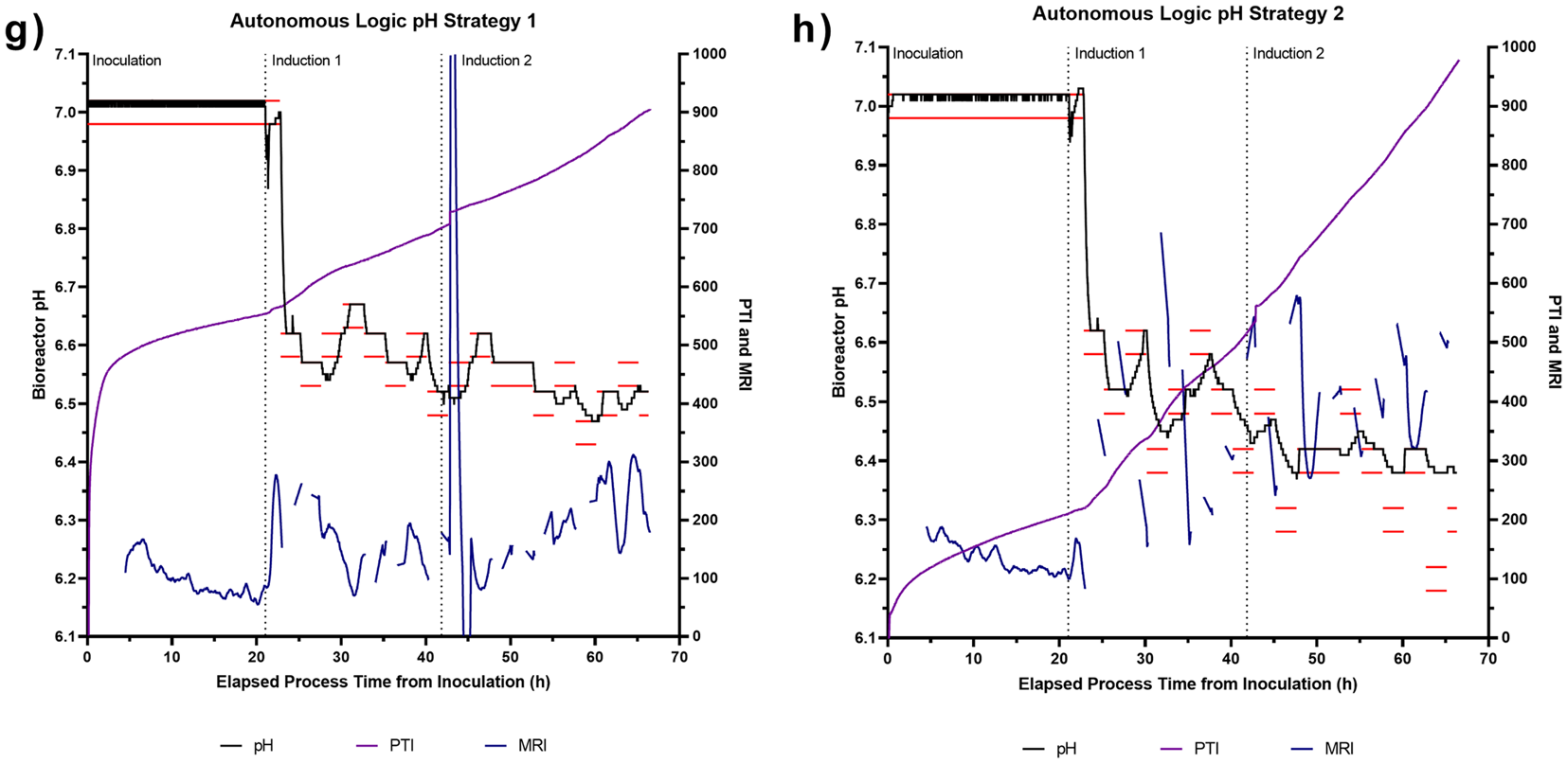
Impact of pre-programmed and autonomous pH parameter control strategies on cell culture activity. (**a**) Pre-programmed pH control strategy 1 and (**b**) strategy 2. PTI and MRI profiles when employing pre-programmed pH control strategy 1 in replicate bioreactors: (**c**) ‘Bioreactor 1’ and (**d**) ‘Bioreactor 2’ and when employing pre-programmed pH control strategy 2 in replicate bioreactors: (**e**) ‘Bioreactor 1’ and (**f**) ‘Bioreactor 2’. (**g**) PTI and MRI traces obtained when employing autonomous pH control strategy 1 and (**h**) strategy 2. In all panels, horizontal red lines indicate pH dead-band and vertical dashed black lines indicate key process events.

Implications of a relationship between bioreactor pH and cellular activity led to the investigation of whether the Ranger RI system could be used to determine and modify the bioreactor pH set-point in a pre-defined and scripted cyclic pattern to establish the relative MRI activities within the cell culture at different stages of the bioprocess. This involved the introduction of periodic pre-programmed changes in the bioreactor pH, to assess how the metabolic activity of the culture would respond to increases and decreases in the environmental pH. Two pre-programmed pH control strategies were investigated. Both strategies employed an initial pH of 6.60, initiated 26 h post-inoculation, and explored different experimental ranges of ± 0.1 pH units and ± 0.2 pH units, denoted as Control Strategy 1 and Control Strategy 2, respectively, from this point forward (Fig. 3a and b). For each strategy, mirrored pH control profiles were investigated to minimise the potential impact of prior cellular processing from the analysis; the rationale being that if an equivalent pH and MRI trend was observed in both mirrored profiles, then the result was not likely influenced by the order of the experiments. It was apparent in all bioreactor systems that a lower bioreactor pH resulted in higher MRI activity with larger differences in activity being observed in the bioreactors running Control Strategy 2 (Fig. 3c–f). In these experiments, each instance of lowering the pH resulted in an increase in MRI, implying that further increases in cellular activity may be attained by lowering the pH further.

To provide further evidence for a relationship between bioreactor pH and MRI activity, an autonomous pH control strategy was employed, which was akin to model predictive control. An initial pH value of 6.60 was defined and the Ranger RI system was then allowed to self-define a pH control protocol that maximised MRI without being provided with any prior process knowledge. This involved the Ranger RI system making real-time assessments of culture MRI, comparing these to historical values within the process and adapting the next pH settings based on this assessment, without any operator intervention. Two autonomous pH control strategies were investigated. Both strategies used an initial pH of 6.60, initiated 26 h post-inoculation, and explored different experimental steps of ± 0.05 pH units and ± 0.10 pH units in the case of Autonomous Strategies 1 and 2, respectively (Fig. 3g and h). The first adjustment in the series is always to a lower pH, but all subsequent adjustments are driven by the cellular process. The bioreactors did not always respond to a set-point change in a timely manner, potentially due to the bioreactor controller’s pH PID loop becoming distorted by the continual rapid changes in pH set-point. However, the Ranger RI system’s autonomous logic works with the achieved pH and not the target set-point so conclusions can still be made in the cases where the pH set-point was not achieved for a particular step. In both experiments, the pH set-point was driven lower than the initial starting pH, with Autonomous Strategy 1 and Autonomous Strategy 2 defining a pH of approximately 6.50 and 6.40, respectively, at the end of the bioprocess. It was anticipated that Strategy 2 would be able to tune pH to maximise MRI activity twice as fast as Strategy 1 due to the larger adjustments that were utilised at part of this approach. In the case of Autonomous Strategy 2, the Ranger RI system attempted to drive the online pH down to a value of 6.20 at the end of the bioprocess. However, the bio-controller was unable to drive the pH below a value of 6.40, possibly due to the pH acidification method employed (i.e., CO_2_ sparging). The results obtained from the processes employing the autonomous control concurred with the conclusions obtained from previous bioreactor experiments employing the pre-programmed pH control strategy, indicating that a bioreactor pH of 6.40 corresponded to the highest MRI activity. This highlighted that the Ranger RI system’s autonomous control logic can be effectively used in place of pre-determined protocols, and without prior process knowledge, in short-duration bioreactor processes. This has the potential to reduce process parameter screening time and cost by negating the need for manual experiments in the future.

### A scripted cell culture recipe designed to maximise metabolic activity was detrimental to lentiviral vector production

**Figure 4:**
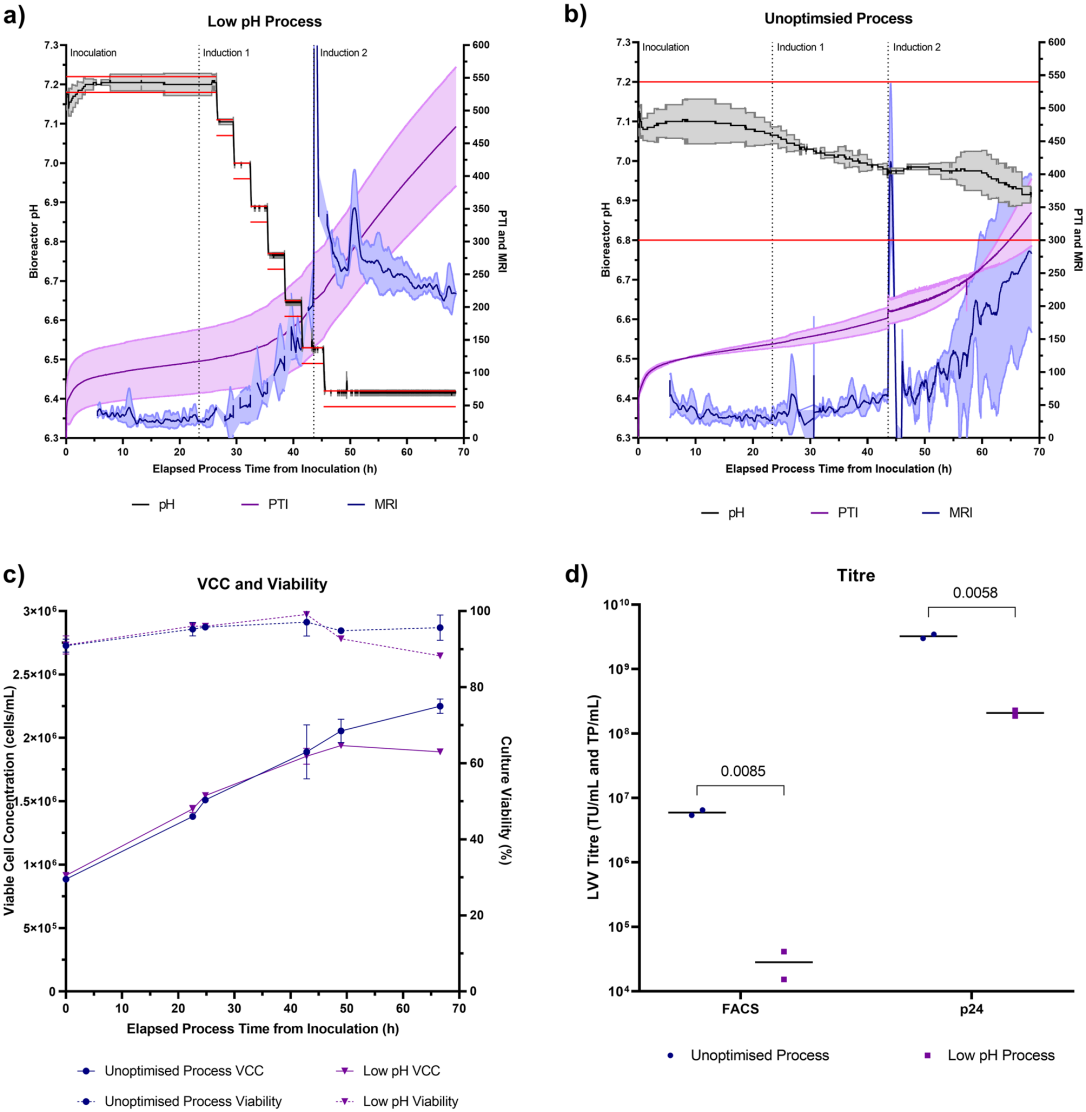
Performance comparison of the ‘low pH’ and ‘unoptimised’ HIV-1-GFP LVV production bioprocesses. PTI, MRI and pH traces obtained when employing the (**a**) ‘Low pH’ control strategy in two replicate bioreactors and when employing the (**b**) ‘Unoptimised’ pH control strategy in two replicate bioreactors. Black, dark purple and dark blue traces indicate the mean of replicate bioreactor processes and grey, light purple and light blue traces indicate ± 1 standard deviation of the mean. Horizontal red lines indicate pH dead-band and vertical dashed black lines indicate key process events. (**c**) VCC and culture viability data obtained from bioreactors employing the two pH control strategies. Data points represent the mean for duplicate bioreactors and errors bars are ± 1 standard deviation of the mean. (**d**) Functional LVV titres and total LVV particles obtained via the two bioprocesses.

The continuous modification of the culture pH throughout the process development experiments did not present favourable conditions to ensure reliable cellular growth and LVV production, due to the rapidly changing culture environment. The process development experiments were used to design a bioprocess that operated a pH set-point of 6.40 ± 0.02 during the LVV production stage, which will henceforth be designated as the ‘low pH’ process; this process attempted to drive higher metabolic activity than that measured in the control experiments. It was hypothesised that boosting metabolic activity, particularly towards the end of the bioprocess, would increase LVV production. Four 5 L bioreactors were used to test this hypothesis: two control processes where pH = 7.00 ± 0.20 for the duration of the bioprocess, in a similar setup to that of the preliminary experiments (henceforth referred to as the ‘unoptimised’ process) and two bioreactors running the ‘low pH’ process (Fig. 4a, b). The ‘low pH’ process started with an initial pH of 7.20 ± 0.02 for the initial 26 h of the process, followed by a gradually stepping down of pH, to avoid a sudden drastic shift in pH that may adversely affect the performance of the cultures, to a set-point of 6.40 ± 0.02 which was maintained from a process time of 46 h. The MRI of the culture increased as a response to the gradual stepdown of pH as expected.

The mean difference in the PTI values that were measured at the onset of the divergence in pH between the two protocols and the end of the bioprocesses, a metric termed 'PTI shift', were calculated as 195 relative units (RU) and 355 RU for the 'unoptimised' and 'low pH' processes, respectively, corresponding to a 1.8-fold increase in metabolic activity in the ‘low pH’ process compared to the ‘unoptimised’ process. Significantly lower average cell concentrations were measured in the ‘low pH’ process compared to the ‘unoptimised’ process at harvest (*p* = 0.0129), likely attributable to the low pH environment not being conducive for cell growth (Fig. 4c). Indeed, it has been suggested that low pH affects cell growth potentially via G1 cell cycle arrest^35^. Despite average culture viabilities of 88.3% and 95.7% being measured in the ‘low pH’ and ‘unoptimised’ processes at harvest, respectively, the difference was not found to be statistically significant (*p* = 0.0895).

The high MRI activity observed in the ‘low pH’ process did not translate into elevated LVV titres (Fig. 4d). A significantly lower concentration of functional LVV particles was measured in samples collected from the ‘low pH’ process, at harvest, than those collected from the ‘unoptimised’ process, with an average titre of 2.83 × 10^4^ TU/mL and 5.95 × 10^6^ TU/mL being calculated, respectively (*p* = 0.0085). The stability of LVV particles at a pH of 6.40 was investigated to determine whether the low titre obtained in the ‘low pH’ process was caused by rapid degradation of functional LVV particles or whether the overall production of vector was indeed low in this process. The total number of LVV particles, determined via p24 ELISA analysis, was calculated to be significantly lower in the samples collected from the ‘low pH’ process than in those collected from the ‘unoptimised’ process (2.09 × 10^8^ particles/mL versus 3.23 × 10^9^ particles/mL, respectively, *p* = 0.0058). This suggested that the low titres were likely not associated with poor vector stability and rapid degradation of functional LVV particles in the pH 6.40 environment but rather, attributable to defective LVV production under these conditions. The elevated metabolic activity observed during cultivation under low pH conditions is likely to be associated with a yet unidentified biochemical process that did not contribute to the processes responsible for LVV production; indeed, our evidence suggests that metabolic resources were actively diverted away from these processes. To deepen our understanding of the metabolic processes between these two conditions, and to suggest a possible explanation for the reduced production of LVV particles in low pH cultures, metabolic modelling analysis was carried out.

### In silico investigation of the metabolic response of HEK293T cells to low pH

Despite causing the cells to increase their metabolic activity, the low pH environment had an adverse effect on LVV production. It was, therefore, desirable to ascertain what the metabolic differences were between the HEK293T-based PCL cultured in low and high pH environments and what drove the increase in MRI, if not LVV production; this was achieved via metabolic flux modelling. The metabolic model was initially configured to mimic the cellular and environmental state of the cells using the limited offline bioprocess information available.

Metabolic models can only reflect specific culture conditions if the model constraints, which represent reaction fluxes, are tuned. In this setting, the exchange fluxes for glucose, lactate, ammonium, sodium, potassium, glutamine and glutamate were computed from the offline parameter measurements. The specific growth rate (*µ*) of the cultures were calculated from the growth profiles as 0.040 h^−1^ for the two ‘unoptimised’ process experiments and as 0.033 h^−1^ and 0.029 h^−1^ for the two ‘low pH’ process experiments (Fig. 4c). The growth rate determined for these cultures were concordant with the literature, where values of 0.036 h^−1^^20^ and 0.029 h^−1^ ^36^ were reported for similar HEK293-based bioprocesses. The specific growth rate of the cultures for which the pH was controlled at a low setpoint was approximately 20% lower than that observed in the ‘unoptimised’ process, indicating that metabolic resources were diverted to support an alternative biological process other than growth in the low pH environment. A cell dry weight (CDW) of 514 pg/cell^37^ was used to determine the dry cell weight of the cultures, and culture-specific exchange fluxes per dry cell weight were calculated using this relationship. The relative differences between the replicates were found to be consistently lower than those between the different process conditions, indicating that the input constraints for the model would be able to assist the identification of the differences between the process runs (Supplementary Table 11).

### Tailoring the model to mimic the metabolic state of the cells in bioprocess cultures

#### Handling of the gluconeogenetic pathway

**Figure 5:**
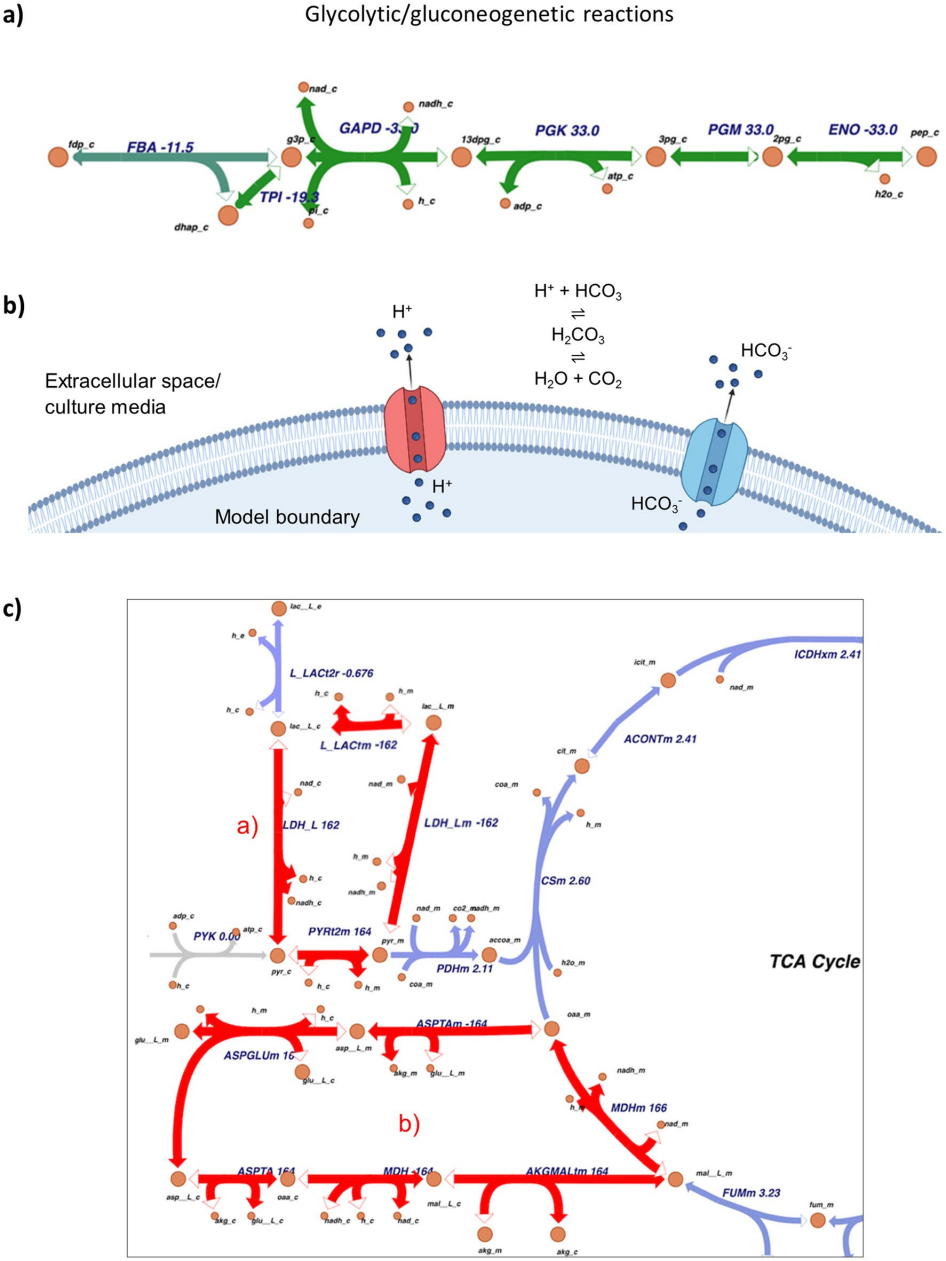
(**a**) Visualisation of six reactions shared by glycolysis and gluconeogenesis. Fluxes were determined by an FBA maximizing biomass production with 1 mmol/(g CDW x h) glucose uptake and all other constraints as default. In this basic state, biomass carbohydrate (synthesized from glucose-6-phosphate) is produced mainly via gluconeogenesis, mostly relying on amino acids supplying the TCA cycle, generating pyruvate, which is then converted into phosphoenolpyruvate. (**b**) Schematic overview of the pH simulation approach. Red: Proton transport channel. Blue: Bicarbonate transport channel. **c:** Extract of the carbon metabolism of validation run 20, highlighting the two high-flux cycles (**a**) and (**b**). It was modelled using the model without sodium and potassium exchange, simulating pH.

Modifications to the model were implemented to design a true representation of the metabolic state of HEK293 cells in their respective cultures. The bioprocess was designed to ensure that the cell cultures never transitioned into a state of glucose starvation at any time point throughout the process. Since activation of the gluconeogenetic pathway was an indicator of the onset of starvation, the availability of the primary carbon source at all times indicated that the gluconeogenetic pathway should not be active for these cells under balanced growth conditions where quasi steady state assumptions would hold. A scenario analysis where the gluconeogenetic pathway was allowed to be utilised in the model indeed showed increased flux through gluconeogenesis despite glucose being available primarily via metabolising amino acids such as L-alanine and L-serine to pyruvate (Fig. 5a), and consequently unrealistic metabolic and growth states were achieved, thus confirming our initial hypothesis.

The metabolic model was modified to accommodate the inhibition of gluconeogenesis in accordance with our hypothesis. This was achieved by allowing flux through all reactions that were shared by glycolysis and gluconeogenesis to run in the glycolytic direction. One exception to this was for the reaction catalysed by triosephosphate isomerase, for which the flux was allowed to run bidirectionally. The flux through the two key reactions of the gluconeogenetic pathway catalysed by phosphoenol-pyruvate carboxykinase and fructose bisphosphatase were constrained to zero. While these modifications drastically improved the predictive capability of the models, further modifications were needed.

#### Sodium and potassium exchange in HEK293 metabolic model

While the original Recon2.2 model included exchange reactions for sodium and potassium, these were missing in the HEK293 metabolic model. Since the concentration of these two cations represent important process parameters used in monitoring the ‘health’ of the cell culture in a typical mammalian bioprocess^38^, the decision was made to include these missing reactions in the current model. Further investigation of the original HEK293 model indicated that while the exchange of these two ions was missing, the model included reactions that utilise these ions. In the absence of their exchange, virtually all reactions that appeared to have utilised these ions were effectively constrained to allow no flux to go through them. This also indicated that the exclusion of these exchange fluxes could perhaps have been mistakenly overlooked historically. Through this modification, we were able to observe differences in the distribution of fluxes at the genome scale, and in particular, were able to restore active flux profiles through those reactions that utilised sodium or potassium ions (Supplementary Table 1).

### Metabolic modelling to elucidate the extracellular pH-driven differences in HEK cell metabolism

The pH differences between the two environmental constraints resulted in differences in the distribution of fluxes. The ‘unoptimised’ process was associated with higher absolute values for the predicted metabolic fluxes than those predicted for the ‘low pH’ process. This observation was accompanied by higher cell growth rates calculated for the ‘unoptimised’ process (Fig. 4c) than those calculated for the ‘low pH’ process.

This difference was particularly prominent for the cyclic interconversion pathways in the metabolic network (Fig. 5c). Furthermore, the differences in pH of the ‘unoptimised’ or the ‘low pH’ processes incorporated into the models provided insight as to how the cells responded to this constraint, as well as providing further insights as to a possible association between pH and the availability of other components for optimal cell growth and metabolic activity. The experimental data showed that the cumulative air inflow in the ‘low pH’ process experiments was approximately twice as high as in the ‘unoptimised’ process, whereas the other gas inflow patterns did not display a similar pattern, in the timeframe of the bioprocess leading to the timepoint at which metabolic modelling was carried out. Our metabolic modelling analysis demonstrated that the carbon dioxide influx was reduced, manifesting itself as elevated extracellular carbon dioxide availability, and the inter-conversion of carbon dioxide and water into carbonic acid, protons and bicarbonate ions through the catalysis of carbonic anhydrase. This reaction was favoured in the direction away from the carbonic acid and bicarbonate production during the ‘low pH’ process with the absolute difference in flux between the ‘low pH’ and the ‘unoptimised’ processes computed as 7.40 and 6.55 mmol/(g CDW × h), respectively. Although the direct oxygen consumption suggested by the modelling was lower in the ‘low pH’ process than the ‘unoptimised’ process, the interconversion between carbon dioxide and bicarbonate that establishes the pH of the extracellular environment at the setpoint provided clues as to the empirically observed elevation in oxygen uptake discussed above. While a significant proportion of the extracellular CO_2_ was being converted into HCO_3_^−^ by the extracellular enzyme, carbonic anhydrase, in the ‘unoptimised’ process, this interconversion was reversed in the ‘low pH’ process contributing to additional production of extracellular CO_2_ in addition to the CO_2_ exported by the cell. This high CO_2_ content is coupled with high dCO_2_ concentration in the ‘low pH’ process, which we hypothesis resulted in a decrease in dO_2_ content via O_2_ stripping. A decrease in dO_2_ would have been detected by the controller and counteracted by increasing the air inflow rate into the culture. It was previously shown that even for aerobic cultures with sustained supply of oxygen, dO_2_ is a limiting factor in operation and the cells cultures can easily suffer from poor dissolved oxygen availability as a result of a shift in the dissolved gas balance of the liquid environment. Stripping by carbon dioxide was reported to be a critical factor affecting the performance of aerobic bioprocesses^39^, and our modelling analysis coupled with the empirical data support this hypothesis.

The metabolic challenge introduced by keeping the cells on the verge of oxygen limitation could explain the bioprocess level observations made: In the low pH cultures (i) the specific growth rate was not able to keep up with the control conditions, (ii) the potential risk and occasionally-observed oxygen limitation triggered stress response leading the upregulation of mechanisms leading to cell death observed as premature loss of viability in these cultures, (iii) the stress described above caused elevated metabolic activity to make the relevant decisions to support survival, and (iv) as a self-preservation strategy, the cell population consequently directed resources away from LVV production, leading to the significantly low productivity. We therefore propose that the elevated metabolic activity, detected by the Ranger RI system, was likely a response to cope with this stress to ensure the maintenance of favourable intracellular conditions. It must be acknowledged that gene regulatory mechanisms could have caused the low levels of LVV production, and the effects observed at the metabolic level could be the manifestation of these hierarchical modifications. This notion simply could not be captured by the combination of available data and the created analysis framework, which mimicked a typical bioprocess development and manufacturing setting.

### Multivariate and univariate regression for building run-specific MRI/PTI soft sensors and univariate forecasting of MRI/PTI

The tight relationship observed between the oxygen availability and acidity of the culture and the mechanistic insight gained from the metabolic modelling analysis as to the possible reasons for these observations then instigated us to turn to bioprocess data to follow the process-level clues left by these metabolic events. This is especially important to understand since the metabolic anomalies and unexpected metabolic activity such as those elucidated by metabolic modelling provide us with in-depth insight, albeit retrospectively. It is of utmost importance to be able to translate this information into bioprocess level understanding in order to be able to detect such irregularities at the time of the process so that relevant interventions could be introduced as required. Our first task to achieve this goal was to look for any associations between MRI, which was already shown empirically to be sensitive to variations in pH, and oxygen availability measured at the bioprocess level, and this was done by feature analysis.

#### Feature analysis to identify correlations and similarities in bioprocess parameters

**Figure 6:**
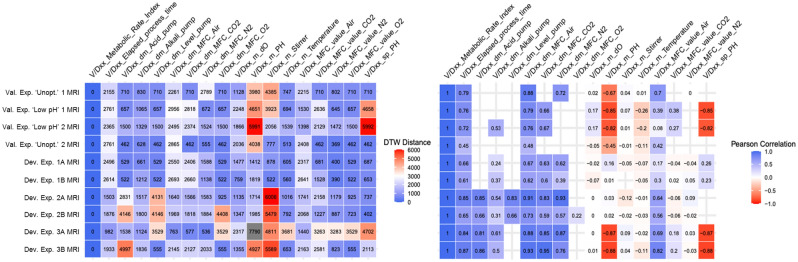
Dynamic time warping distance matrix (left) and Pearson correlation matrix (right). The matrices show the respective similarity metric for the MRI of each run and all online parameters of the same run. DTW distance was capped at approximately 6000, DTW distances exceeding 6000 are displayed in grey. Empty places in the Pearson correlation matrix indicate that the concerned feature did not show any variance, hence, the correlation was mathematically not defined. This figure was generated using R (v. 4.0.3) in RStudio (v. 1.1.463) using the plotting packages “cowplot” (v. 1.1.0) and “ggplot2” (v. 3.3.2). R link: https://mran.microsoft.com/snapshot/2020-12-04/bin/windows/base/. RStudio link: https://www.rstudio.com/products/rstudio/older-versions/#rstudio-desktop-11463.

Similarity (or correlation) between MRI and process parameters was investigated by incorporating complementing insights from two different methods: Dynamic time warping (DTW), a dedicated algorithm to assess the similarity of time series, and Pearson correlation, a measure of the linear correlation of two data series. DTW and Pearson correlation, despite working conceptually very differently, showed similar trends regarding the similarity/correlation of MRI to/with the features for the data used (Fig. 6). There were only two features, which were highly correlated with MRI across all process runs that were analysed, elapsed process time and the cumulative air flow into the bioreactors. Interestingly, process pH did not appear to be consistently correlated with MRI in all of the experiments analysed. This apparent lack of mathematical correlation, while at first sight appeared to contradict the empirical observations, paved the way for the elucidation of a hidden layer of information that interlinks the reduction of pH to an increase in the MRI through oxygen availability monitored through air inflow.

#### Forecasting MRI from process parameters

**Figure 7:**
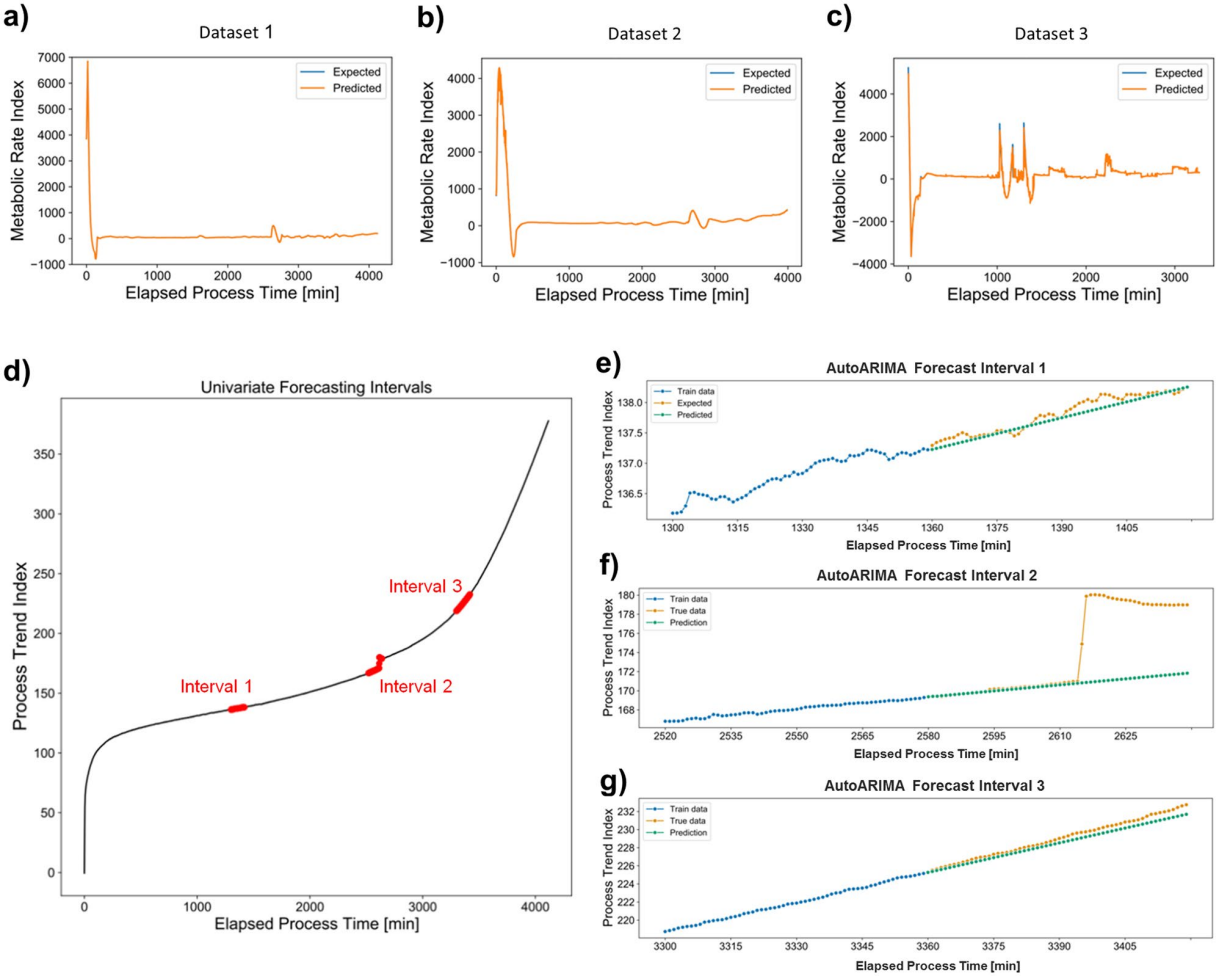
(**a**), (**b**), (**c**) XGBoost Regression models trained and tested on individual datasets. (**a**) RMSE: 12.16, Training set size: 75%. (**b**) RMSE: 29.53, Training set size: 50%. (**c**) RMSE: 102.11, Training set size: 25%. (**d**) PTI curve of ‘unoptimised’ process 1 with the three test intervals for univariate forecasting delineated. (**e**), (**f**), (**g**) AutoARIMA forecasts for the three test intervals. Models were trained on data from the past 60 min and forecasted the next 60 min. RMSEs for entire 60 min forecasts: (**e**) 0.099, (**f**) 5.116, (**g**) 0.544.

Having established that the MRI (or PTI) was representative of a more complex response that did not reliably correlate with other measured bioprocess parameters, indicated that the Ranger RI system could bring in novel insight into the bioprocess, thus acting either to serve as a soft sensor representing the behaviour of other bioprocess parameters not monitored in this work or as an independent sensor in and of itself. To explore this notion further, Artificial Neural Network (ANN)-based approaches were employed, initially to evaluate whether MRI (or PTI) could be forecasted using a computational model that only needs the standard online data as predictor variables. LSTM, a network architecture tailored to handle sequential data was used to carry out this complex univariate forecasting task based on multivariate input. An accurate model was constructed to predict MRI profiles from process variables for each process run with sustained forecasting precision up to a forecasting horizon of two hours even when the training data only covered the past five hours of operation (Supplementary Fig. 5). While the forecasting strategy worked well for individual process runs, a generalisation was not possible due to reasons unclear due to the black box character of LSTM. However, an in-depth understanding of generalisability is essential since a useful soft sensor will need to be broadly applicable and thus key to our question to explore ‘metabolic activity’ as a self-standing process parameter or a soft sensor. Having confirmed that individual processes could indeed be successfully analysed, addressing two separate challenges of carrying out a regression analysis of the multivariate online data and the MRI data followed by the forecasting of MRI to understand the reasons behind the poor generalisability of these models. We then focussed on these challenges separately and the next question we addressed was whether MRI or PTI data could be modelled using regression of the online process data, i.e., whether it would be possible to generate a soft sensor based on the online data to model MRI or PTI in real time. The resulting MRI models consistently performed well at predicting test data from the same process, even when trained on an unusually small training dataset (Fig. 7a–c). Nevertheless, the challenges around generalisability were yet to be resolved. Finally, PTI models were also explored due to the unique features of the PTI datasets. The PTI dataset had virtually no gaps since it was not affected by the system reset after pH set point changes, and second, the trend of PTI was markedly smoother than MRI (Fig. 7d–g). Moreover, the mathematical dependence between the two variables rendered retrospective adjustments for MRI possible in the event that a generalisable working model for PTI could be constructed.

Due to the high positive correlation calculated between PTI and the air flow into the bioreactor through the mass flow controller in all process experiments, univariate forecasting was also explored as an alternative option to multivariate forecasting in this analysis. However, the problems around generalisability were not resolved either for the univariate or multivariate forecasting problems and these results corroborated that there was no consistent underlying relationship between the available online bioprocess parameters and the outputs of the Ranger RI system, MRI and PTI. This was confirmed by an ancillary experiment in which four distinct models, each trained on one dataset, were built, and investigated for the weights assigned to the individual features in each model. The important features were observed to vary for each model, which supported the hypothesis that a consistent relationship between the features and MRI/PTI was absent (Supplementary Fig. 6). This final confirmation concluded the search for generalisability of these models indicating that there were no consistent underlying relationships between the available online bioprocess parameters and the outputs of the Ranger RI system, which could be generalised, rendering the notion that the MRI/PTI could be a soft sensor and that the information provided by the PAT system was indeed new and could not be inferred by modelling standard online bioreactor parameter records.

#### Univariate forecasting of PTI/MRI for system predictability

The final question we addressed was to assess the potential utility of the PAT tool for automated process control. This analysis explored whether past MRI/PTI data could be used to forecast future MRI/PTI reliably. While carrying similar types of information, the smooth trends in PTI data rendered it the preferable candidate for this analysis.

To determine whether univariate forecasting of PTI data could be reliably performed from previous PTI data, three different time intervals were selected in the ‘unoptimised’ process to represent different trends in the data and to ensure a representative impression of the forecasting performance (Fig. 7d). Univariate forecasting, implemented using AutoARIMA from the pmdarima Python library, provided two main insights (Fig. 7e–g). First, steady trends could be forecasted successfully up to one hour into the future. From a process point of view, the implications of this analysis were much wider than expected. Forecasting up to one hour ahead into culture behaviour would allow a sufficient timeframe for an automated system to be able to capture this and to implement necessary control actions in the face of adverse events to reverse the unfavourable process condition observed in cultivation. Secondly, sudden changes in the PTI data, such as that observed in interval two (Fig. 7f) could not be captured, thereby leading to forecasts that were inaccurate. This observation also provides very meaningful insight as to the upstream bioprocess operation. Mammalian systems are usually slow in their metabolic responses, leading to the manifestation of the cognate changes in process parameters in an equally slow mode. The nature of the process in cell and gene therapy applications, unlike, for example, a standard mammalian recombinant protein production process, necessitates manual interventions to be introduced such as the process step for induction of LVV production. These interventions manifested themselves as sudden changes in the PTI data and were unpredictable by the models. As the nature of the change was not inherent to the monitored cellular behaviour or to any of the bioprocess parameters at all, it is very reassuring to see that the models are able to distinguish between these non-native changes and inherent modifications of the cells and the bioprocess during a standard operation.

## Conclusions

There are only a limited number of PAT solutions which are being implemented in upstream advanced therapy medicinal product manufacture at present and the adoption of PAT in the cell and gene therapy (CGT) industry is very much in its infancy. Existing solutions for CGT applications are frequently the same as those used in manufacturing processes for traditional biologics, including sensors for basic process parameters with the analysis of critical quality attributes still being performed offline, placing a limit on their value for improving process control^40^. The novel PAT system used in this work exemplified that the success of an application can be highly context-dependent and may not always ensure the development of improved mammalian bioprocesses. It is important to recognise that many PAT tools were not originally designed specifically for use in viral vector manufacturing applications and will likely need to be optimised and tailored for use in these areas. As the viral vector and wider cell and gene therapy fields mature, they will benefit from both the advances in existing PATs, already developed for the manufacture of conventional biologics, and from the development of novel PAT solutions with specific applications in CGT manufacture^41^. This work demonstrated a trade-off that is widely assumed in the bio-pharmaceutical industry regarding process data acquisition; the collected data suffices to monitor routine manufacturing operations but, if things go wrong, the limited amount of process data cannot always provide immediate and straightforward insights about what failed at the biological level, presenting yet another strong argument for the need for improved PAT. We employed a metabolic flux modelling approach to elucidate the relationship between the observed bioprocess level events and cell metabolism. Since current metabolic network reconstructions do not capture flux regulation in response to extracellular conditions such as pH, an approach circumventing this limitation was devised. To the best of our knowledge, this represents the first time that pH has been thus incorporated into a metabolic model. Using the original model inputs and the pH simulation strategy, metabolic flux modelling provided an explanation for the unusually high air inflow and defective LVV production when attempting to produce LVVs under low pH conditions.

The PAT vision for the biotechnology and biopharmaceutical industries is one that is radical, representing a paradigm shift from a traditional, inflexible, validation-based manufacturing model to one that is based on comprehensive scientific and engineering understanding of bioprocesses. It is evident that a lot more needs to be done to realise this vision and enable the successful delivery of PAT-driven process control strategies. This work exemplifies that there is often not a clear link between critical process parameters (CPPs) and critical quality attributes (CQAs) in such complex systems. Whilst the identification of appropriate CQAs in biopharmaceutical processes is often intuitive, the precise impact of certain CPPs on CQAs is less obvious with the precise relationship only being identified by conducting many experiments. It is, therefore, crucial to continue to evaluate and identify appropriate PAT tools and approaches that will enable the PAT vision and revolutionise biopharmaceutical manufacturing.

## Supplementary Information

Supplementary Information.

## Data Availability

The data that support the findings of this study are included in the Article and its Supplementary Information or are available from the corresponding author upon reasonable request.
