# Supplementary material for: Machine learning and metabolic modelling assisted implementation of a novel process analytical technology in cell and gene therapy manufacturing
Source: Sci Rep. 2023 Jan 16;13:834. doi: 10.1038/s41598-023-27998-2 (PMC9842697; doi:10.1038/s41598-023-27998-2)
Supplement: Supplementary file 1 — Supplementary Information. [file 41598_2023_27998_MOESM1_ESM.docx]

**Supplementary Figures and Tables**

**Supplementary Table 1:** Overview of all reactions for which the addition of sodium and potassium exchange reactions impacted the resulting fluxes in two model-comparing FBAs. The FBAs were optimizing biomass production with gluconeogenesis blocked, fixed constraints for measured metabolites from Run 1 of the ‘unoptimised’ process, all amino acids constrained between -1 and +1 mmol/(g CDW x h) and constraints of ornithine, CO_2_, HCO_3_ and hydrosulfide constrained to zero uptake.

| **Reaction Name** | **Reaction Formula** | **Absolute flux difference** [mmol/(g CDW x h)] | **Flux original**  **model**  [mmol/(g CDW x h)] | **Flux extended model**  [mmol/(g CDW x h)] |
| --- | --- | --- | --- | --- |
| L-lactate dehydrogenase | lac_L[c] + nad[c] <=> pyr[c] + nadh[c] + h[c] | 48.9667 | 50.013 | 1.04623 |
| malate dehydrogenase, mitochondrial | mal_L[m] + nad[m] <=> h[m] + oaa[m] + nadh[m] | 48.9667 | 51.477 | 2.51023 |
| L-lactate transport, mitochondrial | lac_L[c] + h[c] <=> lac_L[m] + h[m] | 48.9667 | -50.689 | -1.72223 |
| alpha-ketoglutarate/malate transporter | mal_L[c] + akg[m] <=> akg[c] + mal_L[m] | 48.9667 | 48.308 | -0.658714 |
| malate dehydrogenase | mal_L[c] + nad[c] <=> oaa[c] + nadh[c] + h[c] | 48.9667 | -51.5212 | -2.55444 |
| aspartate transaminase | asp_L[m] + akg[m] <=> glu_L[m] + oaa[m] | 48.9667 | -48.9667 | 0 |
| L-lactate dehydrogenase | lac_L[m] + nad[m] <=> pyr[m] + h[m] + nadh[m] | 48.9667 | -50.689 | -1.72223 |
| pyruvate mitochondrial transport via proton symport | pyr[c] + h[c] -> pyr[m] + h[m] | 48.9667 | 51.397 | 2.4303 |
| aspartate-glutamate mitochondrial shuttle | glu_L[c] + asp_L[m] + h[i] -> asp_L[c] + glu_L[m] + h[m] | 48.9667 | 48.9667 | 0 |
| aspartate transaminase | asp_L[c] + akg[c] <=> oaa[c] + glu_L[c] | 48.9667 | 49.5306 | 0.563815 |
| carboxylic acid dissociation | co2[c] + h2o[c] <=> hco3[c] + h[c] | 36.7251 | 12.2663 | -24.4587 |
| Carbonic acid hydro-lyase Nitrogen metabolism EC:4.2.1.1 | hco3[e] + h[e] <=> h2o[e] + co2[e] | 36.7251 | 1.61133 | -35.1137 |
| H2O transport via diffusion | h2o[e] <=> h2o[c] | 36.7251 | 0.708274 | -36.0168 |
| CO2 transporter via diffusion | co2[e] <=> co2[c] | 36.7251 | 1.61133 | -35.1137 |
| transport of proline via PAT4 | pro_L[e] <=> pro_L[c] | 24.4834 | 5.80832 | -18.6751 |
| L-proline reversible transport via proton symport | pro_L[e] + h[e] <=> pro_L[c] + h[c] | 24.4834 | -6.62678 | 17.8566 |
| 3HCO3 NAt | 3 hco3[e] + na1[e] <=> 3 hco3[c] + na1[c] | 12.2417 | -2.53118 | 9.7105 |
| KHte | k[e] + h[c] <=> h[e] + k[c] | 12.2417 | 0 | -12.2417 |
| Na+/K+ exchanging ATPase | k[e] + na1[c] + h2o[c] + atp[c] -> na1[e] + pi[c] + adp[c] + k[c] + h[c] | 12.2417 | 0 | 12.2417 |
| ATP synthase (four protons for one ATP) | pi[m] + adp[m] + 4 h[i] -> h2o[m] + atp[m] + 3 h[m] | 12.2417 | 14.024 | 26.2657 |
| ADP/ATP transporter, mitochondrial | adp[c] + atp[m] -> atp[c] + adp[m] | 12.2417 | 14.0604 | 26.3021 |
| phosphate transporter, mitochondrial | pi[c] + h[c] -> pi[m] + h[m] | 12.2417 | 14.1396 | 26.3812 |
| H2O transport, mitochondrial | h2o[c] <=> h2o[m] | 12.2417 | -19.4349 | -31.6766 |

**Supplementary Table 2:** Overview of all reactions for which fluxes differed between two FBAs simulating pH of 6 and 7 respectively. The FBAs were implemented using the extended model containing the Na^+^ and K^+^ exchange reactions. The input flux constraints were prior estimated based on Run 1 of the ‘unoptimised’ process by FVA and then used for both FBAs, only varying HCO_3_ export. FBAs were optimized for biomass production.

| **Reaction Name** | **Reaction Formula** | **Absolute flux difference** [mmol/(g CDW x h)] |
| --- | --- | --- |
| H2O transport via diffusion | h2o[e] <=> h2o[c] | 1.52E-04 |
| L-proline reversible transport via proton symport | pro_L[e] + h[e] <=> pro_L[c] + h[c] | 1.02E-04 |
| carboxylic acid dissociation | co2[c] + h2o[c] <=> hco3[c] + h[c] | 1.02E-04 |
| Carbonic acid hydro-lyase Nitrogen metabolism EC:4.2.1.1 | hco3[e] + h[e] <=> h2o[e] + co2[e] | 1.02E-04 |
| CO2 transporter via diffusion | co2[e] <=> co2[c] | 1.02E-04 |
| 3HCO3 NAt | 3 hco3[e] + na1[e] <=> 3 hco3[c] + na1[c] | 5.08E-05 |
| H2O transport, mitochondrial | h2o[c] <=> h2o[m] | 5.08E-05 |
| L-lactate dehydrogenase | lac_L[c] + nad[c] <=> pyr[c] + nadh[c] + h[c] | 5.08E-05 |
| L-alanine transaminase | ala_L[c] + akg[c] <=> pyr[c] + glu_L[c] | 5.08E-05 |
| H2O exchange | h2o[e] <=> | 5.08E-05 |
| Major Facilitator(MFS) TCDB:2.A.18.6.3 | asp_L[e] + na1[e] -> asp_L[c] + na1[c] | 5.08E-05 |
| L-Aspartate exchange | asp_L[e] <=> | 5.08E-05 |
| Mitochondrial Carrier (MC) TCDB:2.A.29.2.7 | akg[c] + pi[m] -> pi[c] + akg[m] | 5.08E-05 |
| phosphate transporter, mitochondrial | pi[c] + h[c] -> pi[m] + h[m] | 5.08E-05 |
| malic enzyme (NAD), mitochondrial | mal_L[m] + nad[m] -> pyr[m] + co2[m] + nadh[m] | 5.08E-05 |
| exchange reaction for L-alanine | ala_L[e] <=> | 5.08E-05 |
| Bicarbonate exchange | hco3[e] -> | 5.08E-05 |
| aspartate transaminase | asp_L[c] + akg[c] <=> oaa[c] + glu_L[c] | 5.08E-05 |
| carboxylic acid dissociation | h2o[m] + co2[m] <=> hco3[m] + h[m] | 5.08E-05 |
| L-lactate transport, mitochondrial | lac_L[c] + h[c] <=> lac_L[m] + h[m] | 5.08E-05 |
| transport of proline via PAT4 | pro_L[e] <=> pro_L[c] | 5.08E-05 |
| alpha-ketoglutarate/malate transporter | mal_L[c] + akg[m] <=> akg[c] + mal_L[m] | 5.08E-05 |
| Free diffusion | hco3[c] <=> hco3[m] | 5.08E-05 |
| malate dehydrogenase | mal_L[c] + nad[c] <=> oaa[c] + nadh[c] + h[c] | 5.08E-05 |
| Amino Acid-Polyamine-Organocation (APC) TCDB:2.A.3.8.1 | ala_L[e] + pro_L[c] <=> pro_L[e] + ala_L[c] | 5.08E-05 |
| L-lactate dehydrogenase | lac_L[m] + nad[m] <=> pyr[m] + h[m] + nadh[m] | 5.08E-05 |

**Supplementary Table 3:** Overview of the top 10% most different reactions from the FBA solutions with respect to the absolute flux differences between Run 1 of the ‘unoptimised’ process and Run 1 of the ‘low pH’ process. The FBAs were implemented using the extended model **containing the Na^+^ and K^+^ exchange** reactions, and **pH was actively simulated**. The input flux constraints were prior estimated by FVA. FBAs were optimized for biomass production.

| **Reaction Name** | **Reaction Formula** | **Absolute flux difference**  [mmol/(g CDW x h)] | **Flux ‘Unopt.’ R1**  [mmol/(g CDW x h)] | **Flux ‘Low pH’ R1**  [mmol/(g CDW x h)] |
| --- | --- | --- | --- | --- |
| CO2 transporter via diffusion | co2[e] <=> co2[c] | 18.471 | -134.448 | -115.978 |
| H2O transport via diffusion | h2o[e] <=> h2o[c] | 18.388 | -134.024 | -115.636 |
| carboxylic acid dissociation | co2[c] + h2o[c] <=> hco3[c] + h[c] | 17.246 | -126.384 | -109.138 |
| Carbonic acid hydro-lyase Nitrogen metabolism EC:4.2.1.1 | hco3[e] + h[e] <=> h2o[e] + co2[e] | 17.213 | -126.195 | -108.982 |
| L-proline reversible transport via proton symport | pro_L[e] + h[e] <=> pro_L[c] + h[c] | 11.218 | 82.869 | 71.651 |
| transport of proline via PAT4 | pro_L[e] <=> pro_L[c] | 11.201 | -83.690 | -72.489 |
| H2O transport, mitochondrial | h2o[c] <=> h2o[m] | 6.907 | -50.026 | -43.119 |
| Na+/K+ exchanging ATPase | k[e] + na1[c] + h2o[c] + atp[c] -> na1[e] + pi[c] + adp[c] + k[c] + h[c] | 6.086 | 43.844 | 37.758 |
| KHte | k[e] + h[c] <=> h[e] + k[c] | 6.086 | -43.844 | -37.758 |
| ADP/ATP transporter, mitochondrial | adp[c] + atp[m] -> atp[c] + adp[m] | 5.894 | 43.150 | 37.256 |
| phosphate transporter, mitochondrial | pi[c] + h[c] -> pi[m] + h[m] | 5.885 | 44.015 | 38.130 |
| 3HCO3 NAt | 3 hco3[e] + na1[e] <=> 3 hco3[c] + na1[c] | 5.731 | 42.032 | 36.301 |
| ATP synthase (four protons for one ATP) | pi[m] + adp[m] + 4 h[i] -> h2o[m] + atp[m] + 3 h[m] | 5.503 | 40.899 | 35.396 |
| ubiquinol-6 cytochrome c reductase, Complex III | q10h2[m] + 2 ficytC[m] + 2 h[m] -> q10[m] + 2 focytC[m] + 4 h[i] | 2.340 | 18.051 | 15.711 |
| NADH dehydrogenase, mitochondrial | 5 h[m] + nadh[m] + q10[m] -> q10h2[m] + nad[m] + 4 h[i] | 1.955 | 13.841 | 11.886 |
| CO2 exchange | co2[e] -> | 1.257 | 8.254 | 6.996 |
| CO2 transport (diffusion), mitochondrial | co2[c] <=> co2[m] | 1.194 | -7.889 | -6.695 |
| H2O exchange | h2o[e] <=> | 1.174 | 7.829 | 6.655 |
| o2 transport (diffusion) | o2[e] <=> o2[c] | 1.174 | 9.047 | 7.873 |
| exchange reaction for oxugen | o2[e] <=> | 1.174 | -9.047 | -7.873 |
| O2 transport (diffusion) | o2[c] <=> o2[m] | 1.170 | 9.025 | 7.856 |
| cytochrome c oxidase, mitochondrial Complex IV | o2[m] + 8 h[m] + 4 focytC[m] -> 2 h2o[m] + 4 ficytC[m] + 4 h[i] | 1.170 | 9.025 | 7.856 |
| L-lactate dehydrogenase | lac_L[c] + nad[c] <=> pyr[c] + nadh[c] + h[c] | 0.468 | -1.369 | -0.901 |
| phosphoglycerate kinase | 3pg[c] + atp[c] <=> adp[c] + 13dpg[c] | 0.457 | -2.222 | -1.765 |
| phosphoglycerate mutase | 2pg[c] <=> 3pg[c] | 0.457 | -2.222 | -1.765 |
| enolase | 2pg[c] <=> h2o[c] + pep[c] | 0.457 | 2.222 | 1.765 |
| glyceraldehyde-3-phosphate dehydrogenase | pi[c] + nad[c] + g3p[c] <=> 13dpg[c] + nadh[c] + h[c] | 0.457 | 2.222 | 1.765 |
| RE2954 | dtdp[c] + pep[c] + h[c] <=> pyr[c] + dttp[c] | 0.456 | 2.218 | 1.761 |
| nucleoside-diphosphate kinase (ATP:dTDP) | dtdp[c] + atp[c] <=> adp[c] + dttp[c] | 0.456 | -2.217 | -1.761 |
| pyruvate dehydrogenase | pyr[m] + coa[m] + nad[m] -> accoa[m] + co2[m] + nadh[m] | 0.450 | 2.112 | 1.662 |
| citrate synthase | h2o[m] + accoa[m] + oaa[m] -> cit[m] + h[m] + coa[m] | 0.447 | 2.595 | 2.149 |
| Aconitate hydratase | cit[m] <=> icit[m] | 0.415 | 2.414 | 2.000 |
| Isocitrate dehydrogenase (NAD+) | icit[m] + nad[m] -> co2[m] + nadh[m] + akg[m] | 0.415 | 2.414 | 2.000 |
| 2-oxoglutarate dehydrogenase | coa[m] + akg[m] + nad[m] -> succoa[m] + co2[m] + nadh[m] | 0.392 | 2.251 | 1.859 |
| fumarase, mitochondrial | h2o[m] + fum[m] <=> mal_L[m] | 0.388 | 3.233 | 2.844 |
| Succinate-CoA ligase (ADP-forming) | atp[m] + coa[m] + succ[m] <=> pi[m] + adp[m] + succoa[m] | 0.387 | -3.225 | -2.838 |

**Supplementary Table 4:** Overview of the top 10% most different reactions from the FBA solutions with respect to the absolute flux differences between Run 1 of the ‘unoptimised’ process and Run 1 of the ‘low pH’ process. The FBAs were implemented using the extended model **containing the Na^+^ and K^+^ exchange** reactions, and **without** **pH simulation**. The input flux constraints were prior estimated by FVA. FBAs were optimized for biomass production.

| **Reaction Name** | **Reaction Formula** | **Absolute flux difference**  [mmol/(g CDW x h)] | **Flux ‘Unopt.’ R1**  [mmol/(g CDW x h)] | **Flux ‘Low pH’ R1**  [mmol/(g CDW x h)] |
| --- | --- | --- | --- | --- |
| H2O transport via diffusion | h2o[e] <=> h2o[c] | 18.727 | -135.903 | -117.176 |
| Carbonic acid hydro-lyase Nitrogen metabolism EC:4.2.1.1 | hco3[e] + h[e] <=> h2o[e] + co2[e] | 18.697 | -135.701 | -117.004 |
| CO2 transporter via diffusion | co2[e] <=> co2[c] | 18.697 | -135.701 | -117.004 |
| carboxylic acid dissociation | co2[c] + h2o[c] <=> hco3[c] + h[c] | 17.472 | -127.637 | -110.165 |
| L-proline reversible transport via proton symport | pro_L[e] + h[e] <=> pro_L[c] + h[c] | 11.445 | 84.122 | 72.677 |
| transport of proline via PAT4 | pro_L[e] <=> pro_L[c] | 11.314 | -84.316 | -73.002 |
| H2O transport, mitochondrial | h2o[c] <=> h2o[m] | 7.020 | -50.652 | -43.632 |
| KHte | k[e] + h[c] <=> h[e] + k[c] | 6.086 | -43.844 | -37.758 |
| Na+/K+ exchanging ATPase | k[e] + na1[c] + h2o[c] + atp[c] -> na1[e] + pi[c] + adp[c] + k[c] + h[c] | 6.086 | 43.844 | 37.758 |
| ADP/ATP transporter, mitochondrial | adp[c] + atp[m] -> atp[c] + adp[m] | 5.894 | 43.150 | 37.256 |
| 3HCO3 NAt | 3 hco3[e] + na1[e] <=> 3 hco3[c] + na1[c] | 5.844 | 42.658 | 36.814 |
| phosphate transporter, mitochondrial | pi[c] + h[c] -> pi[m] + h[m] | 5.772 | 43.389 | 37.617 |
| ATP synthase (four protons for one ATP) | pi[m] + adp[m] + 4 h[i] -> h2o[m] + atp[m] + 3 h[m] | 5.503 | 40.899 | 35.396 |
| ubiquinol-6 cytochrome c reductase, Complex III | q10h2[m] + 2 ficytC[m] + 2 h[m] -> q10[m] + 2 focytC[m] + 4 h[i] | 2.340 | 18.051 | 15.711 |
| NADH dehydrogenase, mitochondrial | 5 h[m] + nadh[m] + q10[m] -> q10h2[m] + nad[m] + 4 h[i] | 1.955 | 13.841 | 11.886 |
| exchange reaction for proton | h[e] <=> | 1.257 | 8.354 | 7.096 |
| CO2 transport (diffusion), mitochondrial | co2[c] <=> co2[m] | 1.194 | -7.889 | -6.695 |
| o2 transport (diffusion) | o2[e] <=> o2[c] | 1.174 | 9.047 | 7.873 |
| exchange reaction for oxugen | o2[e] <=> | 1.174 | -9.047 | -7.873 |
| cytochrome c oxidase, mitochondrial Complex IV | o2[m] + 8 h[m] + 4 focytC[m] -> 2 h2o[m] + 4 ficytC[m] + 4 h[i] | 1.170 | 9.025 | 7.856 |
| O2 transport (diffusion) | o2[c] <=> o2[m] | 1.170 | 9.025 | 7.856 |
| Bicarbonate exchange | hco3[e] -> | 1.164 | 7.727 | 6.563 |
| L-lactate dehydrogenase | lac_L[c] + nad[c] <=> pyr[c] + nadh[c] + h[c] | 0.582 | -1.995 | -1.414 |
| L-lactate transport, mitochondrial | lac_L[c] + h[c] <=> lac_L[m] + h[m] | 0.481 | 1.319 | 0.839 |
| L-lactate dehydrogenase | lac_L[m] + nad[m] <=> pyr[m] + h[m] + nadh[m] | 0.481 | 1.319 | 0.839 |
| phosphoglycerate kinase | 3pg[c] + atp[c] <=> adp[c] + 13dpg[c] | 0.457 | -2.222 | -1.765 |
| phosphoglycerate mutase | 2pg[c] <=> 3pg[c] | 0.457 | -2.222 | -1.765 |
| enolase | 2pg[c] <=> h2o[c] + pep[c] | 0.457 | 2.222 | 1.765 |
| glyceraldehyde-3-phosphate dehydrogenase | pi[c] + nad[c] + g3p[c] <=> 13dpg[c] + nadh[c] + h[c] | 0.457 | 2.222 | 1.765 |
| RE2954 | dtdp[c] + pep[c] + h[c] <=> pyr[c] + dttp[c] | 0.456 | 2.218 | 1.761 |
| nucleoside-diphosphate kinase (ATP:dTDP) | dtdp[c] + atp[c] <=> adp[c] + dttp[c] | 0.456 | -2.217 | -1.761 |
| pyruvate dehydrogenase | pyr[m] + coa[m] + nad[m] -> accoa[m] + co2[m] + nadh[m] | 0.450 | 2.112 | 1.662 |
| citrate synthase | h2o[m] + accoa[m] + oaa[m] -> cit[m] + h[m] + coa[m] | 0.447 | 2.595 | 2.149 |
| Aconitate hydratase | cit[m] <=> icit[m] | 0.415 | 2.414 | 2.000 |
| Isocitrate dehydrogenase (NAD+) | icit[m] + nad[m] -> co2[m] + nadh[m] + akg[m] | 0.415 | 2.414 | 2.000 |
| 2-oxoglutarate dehydrogenase | coa[m] + akg[m] + nad[m] -> succoa[m] + co2[m] + nadh[m] | 0.392 | 2.251 | 1.859 |

**Supplementary Table 5:** Overview of the top 10% most different reactions from the FBA solutions with respect to the absolute flux differences between Run 1 of the ‘unoptimised’ process and Run 1 of the ‘low pH’ process. The FBAs were implemented using the **original model without the Na^+^ and K^+^ exchange** reactions, and **without** **pH simulation**. The input flux constraints were prior estimated by FVA. FBAs were optimized for biomass production.

| **Reaction Name** | **Reaction Formula** | **Absolute flux difference**  [mmol/(g CDW x h)] | **Flux ‘Unopt.’ R1**  [mmol/(g CDW x h)] | **Flux ‘Low pH’ R1**  [mmol/(g CDW x h)] |
| --- | --- | --- | --- | --- |
| H2O transport via diffusion | h2o[e] <=> h2o[c] | 18.652 | -135.481 | -116.829 |
| Carbonic acid hydro-lyase Nitrogen metabolism EC:4.2.1.1 | hco3[e] + h[e] <=> h2o[e] + co2[e] | 18.622 | -135.280 | -116.658 |
| CO2 transporter via diffusion | co2[e] <=> co2[c] | 18.622 | -135.280 | -116.658 |
| carboxylic acid dissociation | co2[c] + h2o[c] <=> hco3[c] + h[c] | 17.397 | -127.215 | -109.818 |
| L-proline reversible transport via proton symport | pro_L[e] + h[e] <=> pro_L[c] + h[c] | 11.397 | 83.854 | 72.457 |
| transport of proline via PAT4 | pro_L[e] <=> pro_L[c] | 11.274 | -84.089 | -72.815 |
| H2O transport, mitochondrial | h2o[c] <=> h2o[m] | 7.000 | -50.538 | -43.538 |
| Na+/K+ exchanging ATPase | k[e] + na1[c] + h2o[c] + atp[c] -> na1[e] + pi[c] + adp[c] + k[c] + h[c] | 6.062 | 43.710 | 37.648 |
| KHte | k[e] + h[c] <=> h[e] + k[c] | 6.062 | -43.710 | -37.648 |
| ADP/ATP transporter, mitochondrial | adp[c] + atp[m] -> atp[c] + adp[m] | 5.871 | 43.016 | 37.146 |
| 3HCO3 NAt | 3 hco3[e] + na1[e] <=> 3 hco3[c] + na1[c] | 5.820 | 42.524 | 36.704 |
| phosphate transporter, mitochondrial | pi[c] + h[c] -> pi[m] + h[m] | 5.755 | 43.294 | 37.539 |
| ATP synthase (four protons for one ATP) | pi[m] + adp[m] + 4 h[i] -> h2o[m] + atp[m] + 3 h[m] | 5.482 | 40.785 | 35.303 |
| ubiquinol-6 cytochrome c reductase, Complex III | q10h2[m] + 2 ficytC[m] + 2 h[m] -> q10[m] + 2 focytC[m] + 4 h[i] | 2.333 | 18.011 | 15.678 |
| NADH dehydrogenase, mitochondrial | 5 h[m] + nadh[m] + q10[m] -> q10h2[m] + nad[m] + 4 h[i] | 1.948 | 13.802 | 11.853 |
| exchange reaction for proton | h[e] <=> | 1.254 | 8.334 | 7.080 |
| CO2 transport (diffusion), mitochondrial | co2[c] <=> co2[m] | 1.190 | -7.869 | -6.679 |
| o2 transport (diffusion) | o2[e] <=> o2[c] | 1.170 | 9.027 | 7.857 |
| exchange reaction for oxugen | o2[e] <=> | 1.170 | -9.027 | -7.857 |
| cytochrome c oxidase, mitochondrial Complex IV | o2[m] + 8 h[m] + 4 focytC[m] -> 2 h2o[m] + 4 ficytC[m] + 4 h[i] | 1.166 | 9.005 | 7.839 |
| O2 transport (diffusion) | o2[c] <=> o2[m] | 1.166 | 9.005 | 7.839 |
| Bicarbonate exchange | hco3[e] -> | 1.161 | 7.707 | 6.547 |
| L-lactate dehydrogenase | lac_L[c] + nad[c] <=> pyr[c] + nadh[c] + h[c] | 0.575 | -1.956 | -1.381 |
| L-lactate transport, mitochondrial | lac_L[c] + h[c] <=> lac_L[m] + h[m] | 0.474 | 1.280 | 0.806 |
| L-lactate dehydrogenase | lac_L[m] + nad[m] <=> pyr[m] + h[m] + nadh[m] | 0.474 | 1.280 | 0.806 |
| phosphoglycerate kinase | 3pg[c] + atp[c] <=> adp[c] + 13dpg[c] | 0.457 | -2.222 | -1.765 |
| phosphoglycerate mutase | 2pg[c] <=> 3pg[c] | 0.457 | -2.222 | -1.765 |
| enolase | 2pg[c] <=> h2o[c] + pep[c] | 0.457 | 2.222 | 1.765 |
| glyceraldehyde-3-phosphate dehydrogenase | pi[c] + nad[c] + g3p[c] <=> 13dpg[c] + nadh[c] + h[c] | 0.457 | 2.222 | 1.765 |
| RE2954 | dtdp[c] + pep[c] + h[c] <=> pyr[c] + dttp[c] | 0.456 | 2.218 | 1.761 |
| nucleoside-diphosphate kinase (ATP:dTDP) | dtdp[c] + atp[c] <=> adp[c] + dttp[c] | 0.456 | -2.217 | -1.761 |
| pyruvate dehydrogenase | pyr[m] + coa[m] + nad[m] -> accoa[m] + co2[m] + nadh[m] | 0.446 | 2.092 | 1.646 |
| citrate synthase | h2o[m] + accoa[m] + oaa[m] -> cit[m] + h[m] + coa[m] | 0.443 | 2.575 | 2.132 |
| Isocitrate dehydrogenase (NAD+) | icit[m] + nad[m] -> co2[m] + nadh[m] + akg[m] | 0.411 | 2.395 | 1.983 |
| Aconitate hydratase | cit[m] <=> icit[m] | 0.411 | 2.395 | 1.983 |

**Supplementary Table 6:** Overview of the top 10% most different reactions from the FBA solutions with respect to the absolute flux differences between Run 1 of the ‘Unoptimised’ process and Run 1 of the ‘Low pH’ process. The FBAs were implemented using **the original model without the Na^+^ and K^+^ exchange** reactions, and **pH was actively simulated**. The input flux constraints were prior estimated by FVA. FBAs were optimized for biomass production.

| **Reaction Name** | **Reaction Formula** | **Absolute flux difference**  [mmol/(g CDW x h)] | **Flux ‘Unopt.’ R1**  [mmol/(g CDW x h)] | **Flux ‘Low pH’ R1**  [mmol/(g CDW x h)] |
| --- | --- | --- | --- | --- |
| malate dehydrogenase, mitochondrial | mal_L[m] + nad[m] <=> h[m] + oaa[m] + nadh[m] | 162.567 | 166.265 | 3.698 |
| alpha-ketoglutarate/malate transporter | mal_L[c] + akg[m] <=> akg[c] + mal_L[m] | 161.173 | 164.270 | 3.097 |
| malate dehydrogenase | mal_L[c] + nad[c] <=> oaa[c] + nadh[c] + h[c] | 160.448 | -164.451 | -4.002 |
| aspartate transaminase | asp_L[c] + akg[c] <=> oaa[c] + glu_L[c] | 160.428 | 164.270 | 3.841 |
| aspartate transaminase | asp_L[m] + akg[m] <=> glu_L[m] + oaa[m] | 160.320 | -163.669 | -3.349 |
| aspartate-glutamate mitochondrial shuttle | glu_L[c] + asp_L[m] + h[i] -> asp_L[c] + glu_L[m] + h[m] | 160.320 | 163.669 | 3.349 |
| L-lactate transport, mitochondrial | lac_L[c] + h[c] <=> lac_L[m] + h[m] | 160.100 | -162.904 | -2.803 |
| L-lactate dehydrogenase | lac_L[m] + nad[m] <=> pyr[m] + h[m] + nadh[m] | 160.100 | -162.904 | -2.803 |
| L-lactate dehydrogenase | lac_L[c] + nad[c] <=> pyr[c] + nadh[c] + h[c] | 159.999 | 162.228 | 2.228 |
| pyruvate mitochondrial transport via proton symport | pyr[c] + h[c] -> pyr[m] + h[m] | 159.876 | 163.597 | 3.721 |
| ubiquinol-6 cytochrome c reductase, Complex III | q10h2[m] + 2 ficytC[m] + 2 h[m] -> q10[m] + 2 focytC[m] + 4 h[i] | 17.176 | 18.051 | 0.874 |
| NADH dehydrogenase, mitochondrial | 5 h[m] + nadh[m] + q10[m] -> q10h2[m] + nad[m] + 4 h[i] | 13.822 | 13.841 | 0.019 |
| H2O transport via diffusion | h2o[e] <=> h2o[c] | 13.587 | -11.326 | 2.260 |
| CO2 transporter via diffusion | co2[e] <=> co2[c] | 13.287 | -11.751 | 1.537 |
| o2 transport (diffusion) | o2[e] <=> o2[c] | 8.604 | 9.047 | 0.443 |
| exchange reaction for oxugen | o2[e] <=> | 8.604 | -9.047 | -0.443 |
| O2 transport (diffusion) | o2[c] <=> o2[m] | 8.588 | 9.025 | 0.437 |
| cytochrome c oxidase, mitochondrial Complex IV | o2[m] + 8 h[m] + 4 focytC[m] -> 2 h2o[m] + 4 ficytC[m] + 4 h[i] | 8.588 | 9.025 | 0.437 |
| carboxylic acid dissociation | co2[c] + h2o[c] <=> hco3[c] + h[c] | 7.594 | -3.687 | 3.907 |
| H2O exchange | h2o[e] <=> | 7.267 | 7.829 | 0.561 |
| CO2 exchange | co2[e] -> | 6.968 | 8.254 | 1.285 |
| H2O transport, mitochondrial | h2o[c] <=> h2o[m] | 6.699 | -9.127 | -2.428 |
| CO2 transport (diffusion), mitochondrial | co2[c] <=> co2[m] | 6.415 | -7.889 | -1.474 |
| Carbonic acid hydro-lyase Nitrogen metabolism EC:4.2.1.1 | hco3[e] + h[e] <=> h2o[e] + co2[e] | 6.319 | -3.497 | 2.822 |
| L-proline reversible transport via proton symport | pro_L[e] + h[e] <=> pro_L[c] + h[c] | 3.956 | 1.071 | -2.885 |
| glutamate dehydrogenase (NAD) (mitochondrial) | glu_L[m] + h2o[m] + nad[m] <=> nh4[m] + h[m] + nadh[m] + akg[m] | 3.779 | 1.977 | -1.802 |
| electron transfer flavoprotein | fadh2[m] + etfox[m] -> etfrd[m] + fad[m] | 3.353 | 4.205 | 0.851 |
| Electron transfer flavoprotein-ubiquinone oxidoreductase | etfrd[m] + q10[m] -> q10h2[m] + etfox[m] | 3.353 | 4.205 | 0.851 |
| phosphate transporter, mitochondrial | pi[c] + h[c] -> pi[m] + h[m] | 2.837 | 3.116 | 0.278 |
| fumarase, mitochondrial | h2o[m] + fum[m] <=> mal_L[m] | 2.793 | 3.233 | 0.440 |
| Succinate-CoA ligase (ADP-forming) | atp[m] + coa[m] + succ[m] <=> pi[m] + adp[m] + succoa[m] | 2.791 | -3.225 | -0.434 |
| succinate dehydrogenase | fad[m] + succ[m] <=> fadh2[m] + fum[m] | 2.791 | 3.225 | 0.434 |
| transport of proline via PAT4 | pro_L[e] <=> pro_L[c] | 2.776 | -1.892 | 0.885 |
| Na+/K+ exchanging ATPase | k[e] + na1[c] + h2o[c] + atp[c] -> na1[e] + pi[c] + adp[c] + k[c] + h[c] | 2.455 | 2.944 | 0.490 |
| KHte | k[e] + h[c] <=> h[e] + k[c] | 2.455 | -2.944 | -0.490 |

**Supplementary Table 7:** Overview of the top 10% most different reactions from the FBA solutions with respect to the absolute flux differences between Run 2 of the ‘unoptimised’ process and Run 2 of the ‘low pH’ process. The FBAs were implemented using the extended model **containing the Na^+^ and K^+^ exchange** reactions, and **pH was actively simulated**. The input flux constraints were prior estimated by FVA. FBAs were optimized for biomass production.

| **Reaction Name** | **Reaction Formula** | **Absolute flux difference**  [mmol/(g CDW x h)] | **Flux ‘Unopt.’ R2**  [mmol/(g CDW x h)] | **Flux ‘Low pH’ R2**  [mmol/(g CDW x h)] |
| --- | --- | --- | --- | --- |
| CO2 transporter via diffusion | co2[e] <=> co2[c] | 37.371 | -139.692 | -102.321 |
| H2O transport via diffusion | h2o[e] <=> h2o[c] | 37.299 | -139.313 | -102.014 |
| carboxylic acid dissociation | co2[c] + h2o[c] <=> hco3[c] + h[c] | 34.954 | -131.314 | -96.359 |
| Carbonic acid hydro-lyase Nitrogen metabolism EC:4.2.1.1 | hco3[e] + h[e] <=> h2o[e] + co2[e] | 34.931 | -131.155 | -96.225 |
| L-proline reversible transport via proton symport | pro_L[e] + h[e] <=> pro_L[c] + h[c] | 22.820 | 86.141 | 63.321 |
| transport of proline via PAT4 | pro_L[e] <=> pro_L[c] | 22.706 | -86.905 | -64.199 |
| H2O transport, mitochondrial | h2o[c] <=> h2o[m] | 13.936 | -51.975 | -38.040 |
| Na+/K+ exchanging ATPase | k[e] + na1[c] + h2o[c] + atp[c] -> na1[e] + pi[c] + adp[c] + k[c] + h[c] | 12.224 | 45.503 | 33.279 |
| KHte | k[e] + h[c] <=> h[e] + k[c] | 12.224 | -45.503 | -33.279 |
| ADP/ATP transporter, mitochondrial | adp[c] + atp[m] -> atp[c] + adp[m] | 11.821 | 44.755 | 32.933 |
| phosphate transporter, mitochondrial | pi[c] + h[c] -> pi[m] + h[m] | 11.719 | 45.562 | 33.843 |
| 3HCO3 NAt | 3 hco3[e] + na1[e] <=> 3 hco3[c] + na1[c] | 11.633 | 43.685 | 32.052 |
| ATP synthase (four protons for one ATP) | pi[m] + adp[m] + 4 h[i] -> h2o[m] + atp[m] + 3 h[m] | 11.051 | 42.405 | 31.353 |
| ubiquinol-6 cytochrome c reductase, Complex III | q10h2[m] + 2 ficytC[m] + 2 h[m] -> q10[m] + 2 focytC[m] + 4 h[i] | 4.694 | 18.690 | 13.995 |
| NADH dehydrogenase, mitochondrial | 5 h[m] + nadh[m] + q10[m] -> q10h2[m] + nad[m] + 4 h[i] | 3.936 | 14.381 | 10.445 |
| CO2 exchange | co2[e] -> | 2.441 | 8.537 | 6.096 |
| CO2 transport (diffusion), mitochondrial | co2[c] <=> co2[m] | 2.369 | -8.203 | -5.834 |
| H2O exchange | h2o[e] <=> | 2.368 | 8.158 | 5.790 |
| o2 transport (diffusion) | o2[e] <=> o2[c] | 2.353 | 9.366 | 7.013 |
| exchange reaction for oxugen | o2[e] <=> | 2.353 | -9.366 | -7.013 |
| cytochrome c oxidase, mitochondrial Complex IV | o2[m] + 8 h[m] + 4 focytC[m] -> 2 h2o[m] + 4 ficytC[m] + 4 h[i] | 2.347 | 9.345 | 6.998 |
| O2 transport (diffusion) | o2[c] <=> o2[m] | 2.347 | 9.345 | 6.998 |
| L-lactate dehydrogenase | lac_L[c] + nad[c] <=> pyr[c] + nadh[c] + h[c] | 0.949 | -1.496 | -0.547 |
| pyruvate dehydrogenase | pyr[m] + coa[m] + nad[m] -> accoa[m] + co2[m] + nadh[m] | 0.890 | 2.226 | 1.336 |
| citrate synthase | h2o[m] + accoa[m] + oaa[m] -> cit[m] + h[m] + coa[m] | 0.885 | 2.709 | 1.824 |
| phosphoglycerate kinase | 3pg[c] + atp[c] <=> adp[c] + 13dpg[c] | 0.844 | -2.292 | -1.448 |
| phosphoglycerate mutase | 2pg[c] <=> 3pg[c] | 0.844 | -2.292 | -1.448 |
| enolase | 2pg[c] <=> h2o[c] + pep[c] | 0.844 | 2.292 | 1.448 |
| glyceraldehyde-3-phosphate dehydrogenase | pi[c] + nad[c] + g3p[c] <=> 13dpg[c] + nadh[c] + h[c] | 0.844 | 2.292 | 1.448 |
| RE2954 | dtdp[c] + pep[c] + h[c] <=> pyr[c] + dttp[c] | 0.843 | 2.288 | 1.445 |
| nucleoside-diphosphate kinase (ATP:dTDP) | dtdp[c] + atp[c] <=> adp[c] + dttp[c] | 0.843 | -2.287 | -1.444 |
| Aconitate hydratase | cit[m] <=> icit[m] | 0.836 | 2.528 | 1.693 |
| Isocitrate dehydrogenase (NAD+) | icit[m] + nad[m] -> co2[m] + nadh[m] + akg[m] | 0.836 | 2.528 | 1.693 |
| L-lactate transport, mitochondrial | lac_L[c] + h[c] <=> lac_L[m] + h[m] | 0.819 | 0.851 | 0.032 |
| L-lactate dehydrogenase | lac_L[m] + nad[m] <=> pyr[m] + h[m] + nadh[m] | 0.819 | 0.851 | 0.032 |
| 2-oxoglutarate dehydrogenase | coa[m] + akg[m] + nad[m] -> succoa[m] + co2[m] + nadh[m] | 0.770 | 2.350 | 1.580 |

**Supplementary Table 8:** Overview of the top 10% most different reactions from the FBA solutions with respect to the absolute flux differences between Run 2 of the ‘unoptimised’ process and Run 2 of the ‘low pH’ process. The FBAs were implemented using the extended model **containing the Na^+^ and K^+^ exchange** reactions, and **without** **pH simulation**. The input flux constraints were prior estimated by FVA. FBAs were optimized for biomass production.

| **Reaction Name** | **Reaction Formula** | **Absolute flux difference**  [mmol/(g CDW x h)] | **Flux ‘Unopt.’ R2**  [mmol/(g CDW x h)] | **Flux ‘Low pH’ R2**  [mmol/(g CDW x h)] |
| --- | --- | --- | --- | --- |
| H2O transport via diffusion | h2o[e] <=> h2o[c] | 37.745 | -141.105 | -103.361 |
| Carbonic acid hydro-lyase Nitrogen metabolism EC:4.2.1.1 | hco3[e] + h[e] <=> h2o[e] + co2[e] | 37.668 | -140.886 | -103.218 |
| CO2 transporter via diffusion | co2[e] <=> co2[c] | 37.668 | -140.886 | -103.218 |
| carboxylic acid dissociation | co2[c] + h2o[c] <=> hco3[c] + h[c] | 35.251 | -132.508 | -97.257 |
| L-proline reversible transport via proton symport | pro_L[e] + h[e] <=> pro_L[c] + h[c] | 23.118 | 87.336 | 64.218 |
| transport of proline via PAT4 | pro_L[e] <=> pro_L[c] | 22.855 | -87.502 | -64.647 |
| H2O transport, mitochondrial | h2o[c] <=> h2o[m] | 14.084 | -52.573 | -38.488 |
| Na+/K+ exchanging ATPase | k[e] + na1[c] + h2o[c] + atp[c] -> na1[e] + pi[c] + adp[c] + k[c] + h[c] | 12.224 | 45.503 | 33.279 |
| KHte | k[e] + h[c] <=> h[e] + k[c] | 12.224 | -45.503 | -33.279 |
| ADP/ATP transporter, mitochondrial | adp[c] + atp[m] -> atp[c] + adp[m] | 11.821 | 44.755 | 32.933 |
| 3HCO3 NAt | 3 hco3[e] + na1[e] <=> 3 hco3[c] + na1[c] | 11.782 | 44.282 | 32.501 |
| phosphate transporter, mitochondrial | pi[c] + h[c] -> pi[m] + h[m] | 11.571 | 44.965 | 33.394 |
| ATP synthase (four protons for one ATP) | pi[m] + adp[m] + 4 h[i] -> h2o[m] + atp[m] + 3 h[m] | 11.051 | 42.405 | 31.353 |
| ubiquinol-6 cytochrome c reductase, Complex III | q10h2[m] + 2 ficytC[m] + 2 h[m] -> q10[m] + 2 focytC[m] + 4 h[i] | 4.694 | 18.690 | 13.995 |
| NADH dehydrogenase, mitochondrial | 5 h[m] + nadh[m] + q10[m] -> q10h2[m] + nad[m] + 4 h[i] | 3.936 | 14.381 | 10.445 |
| exchange reaction for proton | h[e] <=> | 2.441 | 8.637 | 6.196 |
| CO2 transport (diffusion), mitochondrial | co2[c] <=> co2[m] | 2.369 | -8.203 | -5.834 |
| o2 transport (diffusion) | o2[e] <=> o2[c] | 2.353 | 9.366 | 7.013 |
| exchange reaction for oxugen | o2[e] <=> | 2.353 | -9.366 | -7.013 |
| cytochrome c oxidase, mitochondrial Complex IV | o2[m] + 8 h[m] + 4 focytC[m] -> 2 h2o[m] + 4 ficytC[m] + 4 h[i] | 2.347 | 9.345 | 6.998 |
| O2 transport (diffusion) | o2[c] <=> o2[m] | 2.347 | 9.345 | 6.998 |
| Bicarbonate exchange | hco3[e] -> | 2.324 | 8.039 | 5.716 |
| L-lactate dehydrogenase | lac_L[c] + nad[c] <=> pyr[c] + nadh[c] + h[c] | 1.098 | -2.093 | -0.995 |
| L-lactate transport, mitochondrial | lac_L[c] + h[c] <=> lac_L[m] + h[m] | 0.968 | 1.448 | 0.480 |
| L-lactate dehydrogenase | lac_L[m] + nad[m] <=> pyr[m] + h[m] + nadh[m] | 0.968 | 1.448 | 0.480 |
| pyruvate dehydrogenase | pyr[m] + coa[m] + nad[m] -> accoa[m] + co2[m] + nadh[m] | 0.890 | 2.226 | 1.336 |
| citrate synthase | h2o[m] + accoa[m] + oaa[m] -> cit[m] + h[m] + coa[m] | 0.885 | 2.709 | 1.824 |
| phosphoglycerate kinase | 3pg[c] + atp[c] <=> adp[c] + 13dpg[c] | 0.844 | -2.292 | -1.448 |
| phosphoglycerate mutase | 2pg[c] <=> 3pg[c] | 0.844 | -2.292 | -1.448 |
| enolase | 2pg[c] <=> h2o[c] + pep[c] | 0.844 | 2.292 | 1.448 |
| glyceraldehyde-3-phosphate dehydrogenase | pi[c] + nad[c] + g3p[c] <=> 13dpg[c] + nadh[c] + h[c] | 0.844 | 2.292 | 1.448 |
| RE2954 | dtdp[c] + pep[c] + h[c] <=> pyr[c] + dttp[c] | 0.843 | 2.288 | 1.445 |
| nucleoside-diphosphate kinase (ATP:dTDP) | dtdp[c] + atp[c] <=> adp[c] + dttp[c] | 0.843 | -2.287 | -1.444 |
| Aconitate hydratase | cit[m] <=> icit[m] | 0.836 | 2.528 | 1.693 |
| Isocitrate dehydrogenase (NAD+) | icit[m] + nad[m] -> co2[m] + nadh[m] + akg[m] | 0.836 | 2.528 | 1.693 |
| 2-oxoglutarate dehydrogenase | coa[m] + akg[m] + nad[m] -> succoa[m] + co2[m] + nadh[m] | 0.770 | 2.350 | 1.580 |

**Supplementary Table 9:** Overview of the top 10% most different reactions from the FBA solutions with respect to the absolute flux differences between Run 2 of the ‘unoptimised’ process and Run 2 of the ‘low pH’ process. The FBAs were implemented using the **original model without the Na^+^ and K^+^ exchange** reactions, and **without** **pH simulation**. The input flux constraints were prior estimated by FVA. FBAs were optimized for biomass production.

| **Reaction Name** | **Reaction Formula** | **Absolute flux difference**  [mmol/(g CDW x h)] | **Flux ‘Unopt.’ R2**  [mmol/(g CDW x h)] | **Flux ‘Low pH’ R2**  [mmol/(g CDW x h)] |
| --- | --- | --- | --- | --- |
| H2O transport via diffusion | h2o[e] <=> h2o[c] | 37.629 | -140.684 | -103.055 |
| Carbonic acid hydro-lyase Nitrogen metabolism EC:4.2.1.1 | hco3[e] + h[e] <=> h2o[e] + co2[e] | 37.552 | -140.465 | -102.913 |
| CO2 transporter via diffusion | co2[e] <=> co2[c] | 37.552 | -140.465 | -102.913 |
| carboxylic acid dissociation | co2[c] + h2o[c] <=> hco3[c] + h[c] | 35.136 | -132.087 | -96.951 |
| L-proline reversible transport via proton symport | pro_L[e] + h[e] <=> pro_L[c] + h[c] | 23.044 | 87.068 | 64.024 |
| transport of proline via PAT4 | pro_L[e] <=> pro_L[c] | 22.792 | -87.275 | -64.482 |
| H2O transport, mitochondrial | h2o[c] <=> h2o[m] | 14.053 | -52.459 | -38.406 |
| KHte | k[e] + h[c] <=> h[e] + k[c] | 12.188 | -45.369 | -33.182 |
| Na+/K+ exchanging ATPase | k[e] + na1[c] + h2o[c] + atp[c] -> na1[e] + pi[c] + adp[c] + k[c] + h[c] | 12.188 | 45.369 | 33.182 |
| ADP/ATP transporter, mitochondrial | adp[c] + atp[m] -> atp[c] + adp[m] | 11.784 | 44.621 | 32.836 |
| 3HCO3 NAt | 3 hco3[e] + na1[e] <=> 3 hco3[c] + na1[c] | 11.745 | 44.149 | 32.404 |
| phosphate transporter, mitochondrial | pi[c] + h[c] -> pi[m] + h[m] | 11.545 | 44.871 | 33.326 |
| ATP synthase (four protons for one ATP) | pi[m] + adp[m] + 4 h[i] -> h2o[m] + atp[m] + 3 h[m] | 11.020 | 42.291 | 31.271 |
| ubiquinol-6 cytochrome c reductase, Complex III | q10h2[m] + 2 ficytC[m] + 2 h[m] -> q10[m] + 2 focytC[m] + 4 h[i] | 4.683 | 18.650 | 13.967 |
| NADH dehydrogenase, mitochondrial | 5 h[m] + nadh[m] + q10[m] -> q10h2[m] + nad[m] + 4 h[i] | 3.925 | 14.342 | 10.417 |
| exchange reaction for proton | h[e] <=> | 2.435 | 8.617 | 6.182 |
| CO2 transport (diffusion), mitochondrial | co2[c] <=> co2[m] | 2.363 | -8.183 | -5.820 |
| o2 transport (diffusion) | o2[e] <=> o2[c] | 2.348 | 9.346 | 6.999 |
| exchange reaction for oxugen | o2[e] <=> | 2.348 | -9.346 | -6.999 |
| O2 transport (diffusion) | o2[c] <=> o2[m] | 2.342 | 9.325 | 6.983 |
| cytochrome c oxidase, mitochondrial Complex IV | o2[m] + 8 h[m] + 4 focytC[m] -> 2 h2o[m] + 4 ficytC[m] + 4 h[i] | 2.342 | 9.325 | 6.983 |
| Bicarbonate exchange | hco3[e] -> | 2.318 | 8.019 | 5.701 |
| L-lactate dehydrogenase | lac_L[c] + nad[c] <=> pyr[c] + nadh[c] + h[c] | 1.087 | -2.054 | -0.967 |
| L-lactate transport, mitochondrial | lac_L[c] + h[c] <=> lac_L[m] + h[m] | 0.957 | 1.409 | 0.452 |
| L-lactate dehydrogenase | lac_L[m] + nad[m] <=> pyr[m] + h[m] + nadh[m] | 0.957 | 1.409 | 0.452 |
| pyruvate dehydrogenase | pyr[m] + coa[m] + nad[m] -> accoa[m] + co2[m] + nadh[m] | 0.885 | 2.206 | 1.321 |
| citrate synthase | h2o[m] + accoa[m] + oaa[m] -> cit[m] + h[m] + coa[m] | 0.880 | 2.689 | 1.809 |
| phosphoglycerate kinase | 3pg[c] + atp[c] <=> adp[c] + 13dpg[c] | 0.844 | -2.292 | -1.448 |
| phosphoglycerate mutase | 2pg[c] <=> 3pg[c] | 0.844 | -2.292 | -1.448 |
| enolase | 2pg[c] <=> h2o[c] + pep[c] | 0.844 | 2.292 | 1.448 |
| glyceraldehyde-3-phosphate dehydrogenase | pi[c] + nad[c] + g3p[c] <=> 13dpg[c] + nadh[c] + h[c] | 0.844 | 2.292 | 1.448 |
| RE2954 | dtdp[c] + pep[c] + h[c] <=> pyr[c] + dttp[c] | 0.843 | 2.288 | 1.445 |
| nucleoside-diphosphate kinase (ATP:dTDP) | dtdp[c] + atp[c] <=> adp[c] + dttp[c] | 0.843 | -2.287 | -1.444 |
| Isocitrate dehydrogenase (NAD+) | icit[m] + nad[m] -> co2[m] + nadh[m] + akg[m] | 0.830 | 2.509 | 1.678 |
| Aconitate hydratase | cit[m] <=> icit[m] | 0.830 | 2.509 | 1.678 |

**Supplementary Table 10:** Overview of the top 10% most different reactions from the FBA solutions with respect to the absolute flux differences between Run 2 of the ‘unoptimised’ process and Run 2 of the ‘low pH’ process. The FBAs were implemented using **the original model without the Na^+^ and K^+^ exchange** reactions, and **pH was actively simulated**. The input flux constraints were prior estimated by FVA. FBAs were optimized for biomass production.

| **Reaction Name** | **Reaction Formula** | **Absolute flux difference**  [mmol/(g CDW x h)] | **Flux ‘Unopt.’ R2**  [mmol/(g CDW x h)] | **Flux ‘Low pH’ R2**  [mmol/(g CDW x h)] |
| --- | --- | --- | --- | --- |
| malate dehydrogenase, mitochondrial | mal_L[m] + nad[m] <=> h[m] + oaa[m] + nadh[m] | 165.810 | 172.373 | 6.563 |
| alpha-ketoglutarate/malate transporter | mal_L[c] + akg[m] <=> akg[c] + mal_L[m] | 164.325 | 170.235 | 5.909 |
| malate dehydrogenase | mal_L[c] + nad[c] <=> oaa[c] + nadh[c] + h[c] | 163.713 | -170.416 | -6.702 |
| aspartate transaminase | asp_L[c] + akg[c] <=> oaa[c] + glu_L[c] | 163.674 | 170.235 | 6.560 |
| aspartate-glutamate mitochondrial shuttle | glu_L[c] + asp_L[m] + h[i] -> asp_L[c] + glu_L[m] + h[m] | 163.533 | 169.663 | 6.131 |
| aspartate transaminase | asp_L[m] + akg[m] <=> glu_L[m] + oaa[m] | 163.533 | -169.663 | -6.131 |
| pyruvate mitochondrial transport via proton symport | pyr[c] + h[c] -> pyr[m] + h[m] | 163.288 | 169.619 | 6.330 |
| L-lactate transport, mitochondrial | lac_L[c] + h[c] <=> lac_L[m] + h[m] | 163.006 | -168.768 | -5.762 |
| L-lactate dehydrogenase | lac_L[m] + nad[m] <=> pyr[m] + h[m] + nadh[m] | 163.006 | -168.768 | -5.762 |
| L-lactate dehydrogenase | lac_L[c] + nad[c] <=> pyr[c] + nadh[c] + h[c] | 162.876 | 168.123 | 5.247 |
| ubiquinol-6 cytochrome c reductase, Complex III | q10h2[m] + 2 ficytC[m] + 2 h[m] -> q10[m] + 2 focytC[m] + 4 h[i] | 17.550 | 18.690 | 1.139 |
| NADH dehydrogenase, mitochondrial | 5 h[m] + nadh[m] + q10[m] -> q10h2[m] + nad[m] + 4 h[i] | 14.243 | 14.381 | 0.138 |
| H2O transport via diffusion | h2o[e] <=> h2o[c] | 14.188 | -12.099 | 2.089 |
| CO2 transporter via diffusion | co2[e] <=> co2[c] | 13.949 | -12.478 | 1.471 |
| o2 transport (diffusion) | o2[e] <=> o2[c] | 8.792 | 9.366 | 0.574 |
| exchange reaction for oxugen | o2[e] <=> | 8.792 | -9.366 | -0.574 |
| O2 transport (diffusion) | o2[c] <=> o2[m] | 8.775 | 9.345 | 0.570 |
| cytochrome c oxidase, mitochondrial Complex IV | o2[m] + 8 h[m] + 4 focytC[m] -> 2 h2o[m] + 4 ficytC[m] + 4 h[i] | 8.775 | 9.345 | 0.570 |
| carboxylic acid dissociation | co2[c] + h2o[c] <=> hco3[c] + h[c] | 7.657 | -4.100 | 3.557 |
| H2O exchange | h2o[e] <=> | 7.639 | 8.158 | 0.518 |
| H2O transport, mitochondrial | h2o[c] <=> h2o[m] | 7.418 | -9.571 | -2.153 |
| CO2 exchange | co2[e] -> | 7.401 | 8.537 | 1.136 |
| CO2 transport (diffusion), mitochondrial | co2[c] <=> co2[m] | 6.902 | -8.203 | -1.301 |
| Carbonic acid hydro-lyase Nitrogen metabolism EC:4.2.1.1 | hco3[e] + h[e] <=> h2o[e] + co2[e] | 6.549 | -3.941 | 2.607 |
| L-proline reversible transport via proton symport | pro_L[e] + h[e] <=> pro_L[c] + h[c] | 3.899 | 1.332 | -2.567 |
| glutamate dehydrogenase (NAD) (mitochondrial) | glu_L[m] + h2o[m] + nad[m] <=> nh4[m] + h[m] + nadh[m] + akg[m] | 3.548 | 1.946 | -1.602 |
| electron transfer flavoprotein | fadh2[m] + etfox[m] -> etfrd[m] + fad[m] | 3.306 | 4.304 | 0.998 |
| Electron transfer flavoprotein-ubiquinone oxidoreductase | etfrd[m] + q10[m] -> q10h2[m] + etfox[m] | 3.306 | 4.304 | 0.998 |
| fumarase, mitochondrial | h2o[m] + fum[m] <=> mal_L[m] | 2.820 | 3.332 | 0.511 |
| Succinate--CoA ligase (ADP-forming) | atp[m] + coa[m] + succ[m] <=> pi[m] + adp[m] + succoa[m] | 2.818 | -3.324 | -0.506 |
| succinate dehydrogenase | fad[m] + succ[m] <=> fadh2[m] + fum[m] | 2.818 | 3.324 | 0.506 |
| phosphate transporter, mitochondrial | pi[c] + h[c] -> pi[m] + h[m] | 2.789 | 3.158 | 0.369 |
| transport of proline via PAT4 | pro_L[e] <=> pro_L[c] | 2.771 | -2.096 | 0.675 |
| Na+/K+ exchanging ATPase | k[e] + na1[c] + h2o[c] + atp[c] -> na1[e] + pi[c] + adp[c] + k[c] + h[c] | 2.764 | 3.098 | 0.335 |
| KHte | k[e] + h[c] <=> h[e] + k[c] | 2.764 | -3.098 | -0.335 |

**Supplementary Table 11:** Calculated metabolic fluxes of the offline measured metabolites for each validation run, grouped by duplicates, and corresponding means and percentage differences.

| **Process run^*^** | **Glc^**^ flux**  [mmol/(g CDW x h)] | **Lac^**^ flux** [mmol/(g CDW x h)] | **NH_4_^+**^ flux** [mmol/(g CDW x h)] | **Na^+**^ flux** [mmol/(g CDW x h)] | **K^+**^ flux** [mmol/(g CDW x h)] | **Gln^**^ flux** [mmol/(g CDW x h)] | **Glu^**^ flux** [mmol/(g CDW x h)] |
| --- | --- | --- | --- | --- | --- | --- | --- |
| **1** | 1.136 | 0.676 | 0.058 | 6.079 | 0.192 | 0.116 | 0.019 |
| **2** | 1.171 | 0.645 | 0.056 | 6.102 | 0.196 | 0.131 | 0.019 |
| **∅_1_** | 1.153 | 0.660 | 0.057 | 6.090 | 0.194 | 0.123 | 0.019 |
| **Variation [%]** | **3.0%** | **-4.6%** | **-2.9%** | **0.4%** | **1.8%** | **13.3%** | **0.0%** |
| **3** | 0.903 | 0.575 | 0.048 | 4.900 | 0.156 | 0.102 | 0.015 |
| **4** | 0.742 | 0.515 | 0.040 | 4.177 | 0.132 | 0.079 | 0.013 |
| **∅_2_** | 0.823 | 0.545 | 0.044 | 4.539 | 0.144 | 0.091 | 0.014 |
| **Variation [%]** | **-17.8%** | **-10.5%** | **-15.9%** | **-14.7%** | **-15.5%** | **-22.1%** | **-14.3%** |
| **Variation of means [%]** | **-28.68%** | **-17.46%** | **-23.15%** | **-25.48%** | **-25.62%** | **-26.66%** | **-25.77%** |

^*^Runs 1 and 2 and runs 3 and 4 denote the replicate runs for the ‘unoptimised’ process and the ‘low pH’ process, respectively.

^**^Abbreviations: Glc, Lac, NH_4_^+^, Na^+^, K^+^, Gln, Glu denote glucose, lactate, ammonium, sodium, potassium, glutamine and glutamate, respectively.

**Supplementary Table 12:** Summary of the model adjustments for flux variability analysis.

| **Adjusted reaction fluxes** | **Measure taken** | **Rationale** |
| --- | --- | --- |
| Glycolysis | Constrained to allow only unidirectional flux (see chapter 3.2.3.) | Disable gluconeogenesis |
| Gluconeogenesis | Constrained to zero (see chapter 3.2.3.) | Disable gluconeogenesis |
| Biomass production | Fixed to measured specific growth rate | Model close to real conditions |
| Glucose, Lactate, Ammonium exchange | Constrained to fixed computed values (see Table 2) | Model close to real conditions |
| Ornithine, CO_2_, HCO_3_^-^, hydrosulfide exchange | Constrained to zero uptake | Ornithine and hydrosulfide: not present in the culture medium; CO_2_, HCO_3_^-^: to avoid use as alternative carbon source |
| Proton, HCO_3_^-^ exchange | Adjusted to computed values at given pH | Simulate pH (see chapter 3.2.5.) |
| Glutamine, Glutamate | Constrained lower bound to zero, upper bound to computed flux (see Table 2) | Use both as objective functions |

| **a)**  **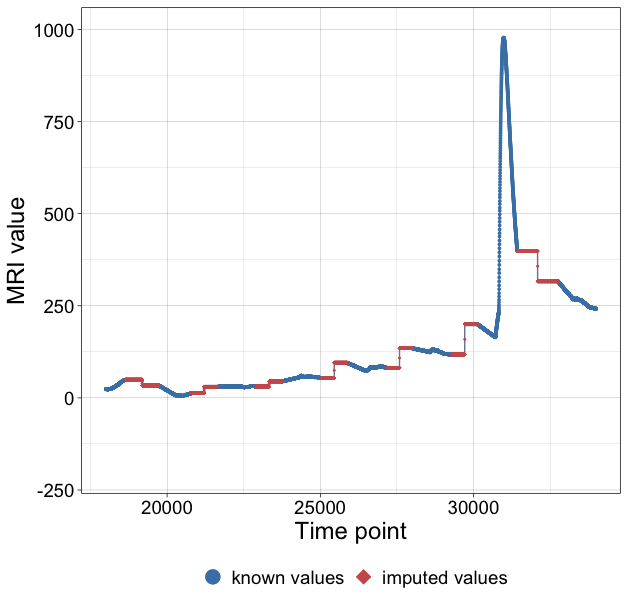** | **b)**  **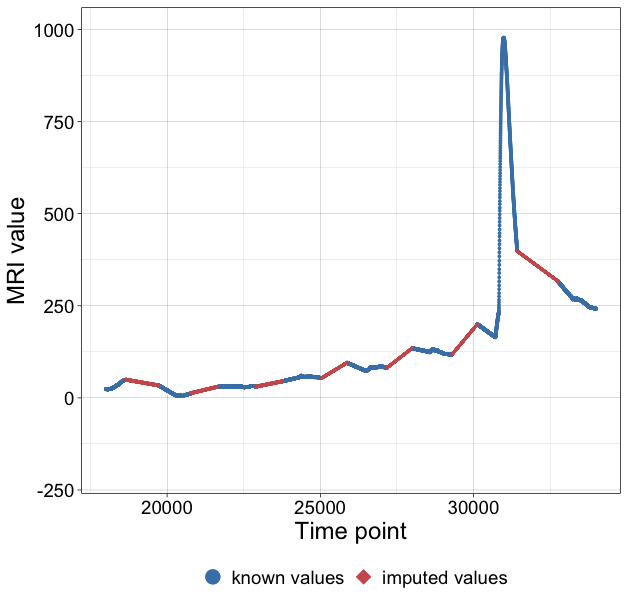** |
| --- | --- |
| **c)**  **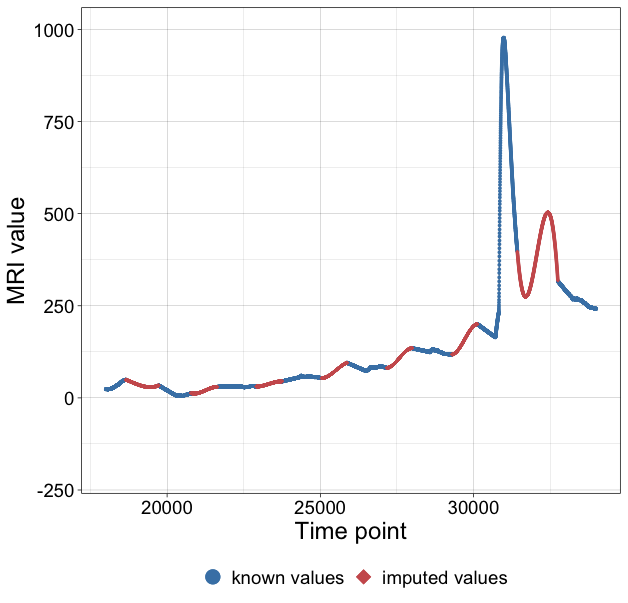** | **d)**  **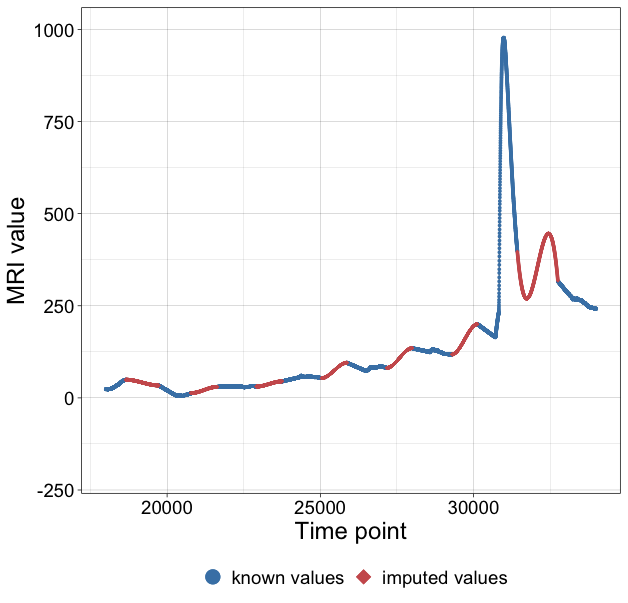** |
| **e)**  **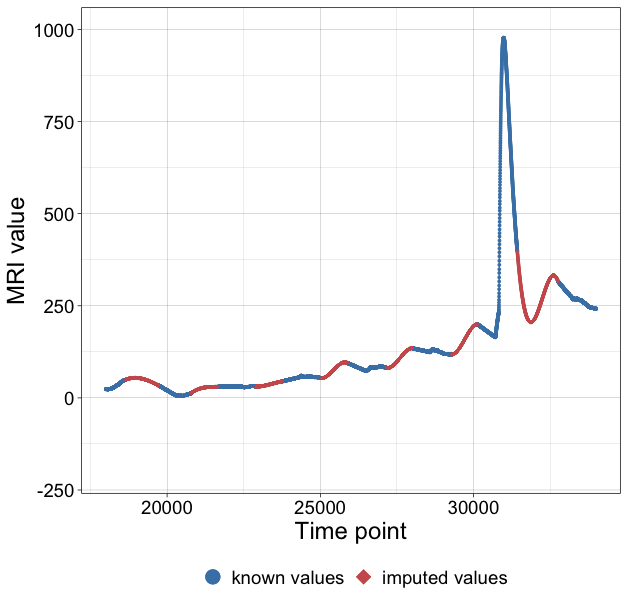** | **f)**  **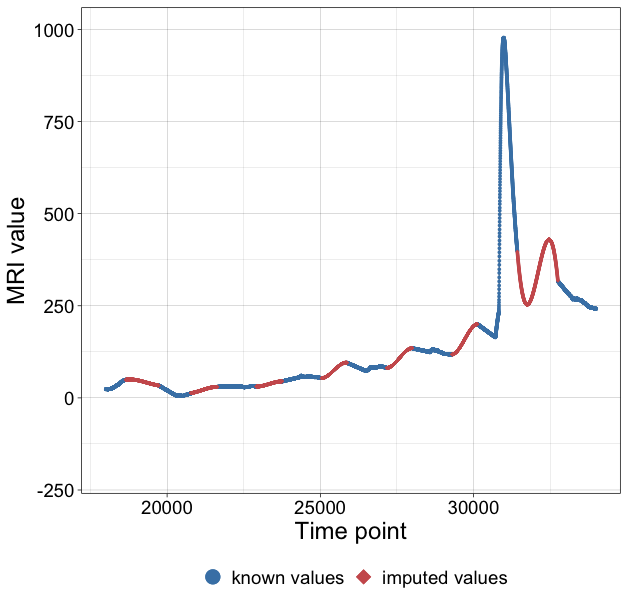** |

**Supplementary Figure 1:** Visualisations of different methods for missing value replacement for the MRI time series data of validation run 19 between data reads 18,000 and 34,000. **a)** Simple moving average. **b)** Linear interpolation **c)** Spline Interpolation **d)** Stineman Interpolation **e)** Kalman smoothing on AutoARIMA. **f)** Kalman smoothing on a structural model. Kalman smoothing on AutoARIMA seems to produce the most natural imputation (Supplementary Figure 2).

|  |
| --- |

**Supplementary Figure 2:** MRI curves of four development runs where no data was missing. The sine-shaped intervals between 40 h and 50 h were used as a reference to assess the gap imputation shown in Supplementary Figure 1. According to this, Kalman smoothing on AutoARIMA was deemed the best method from the imputeTS functions because after the trough of the sine-shape the curve does not rise as high anymore as imputed by Spline interpolation, Stineman interpolation and Kalman smoothing on a structural model.

| **a)**  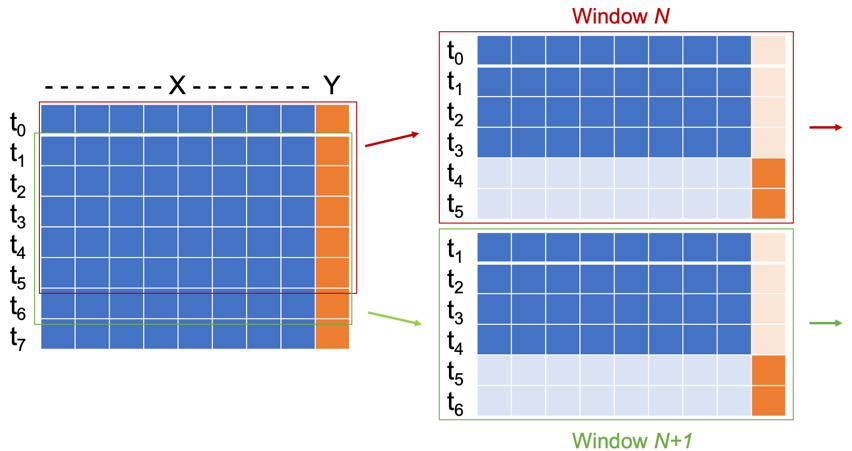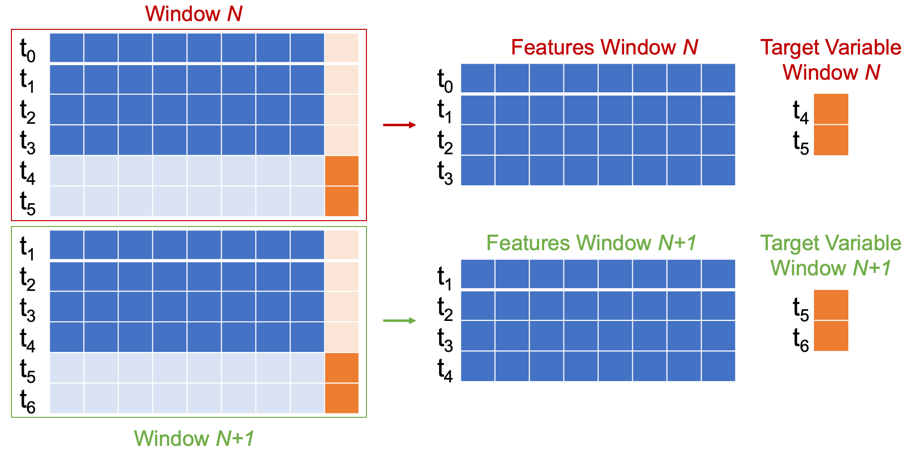 |
| --- |
| **b)**  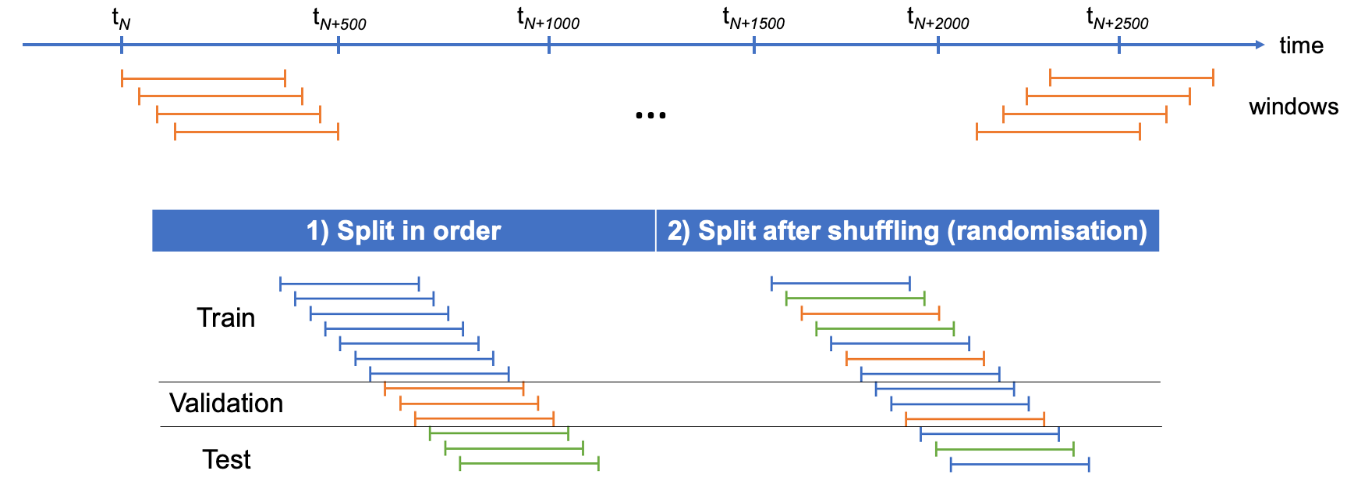 |

**Supplementary Figure 3: a)** Concept of the moving window approach for LSTM training. Initially, two parameters must be set: “n_past”, defining the number of past time points for which the feature values are presented to the model, and “n_future”, defining the forecasting horizon. The resulting modelling task is to learn on “n_past” instances of the features and forecast “n_future” instances of the target variable. Every window covers a time span of “n_past” + “n_future” and subsequent windows are shifted by one time point. **b)** Conceptual difference between the two sampling strategies applied for LSTM training.

| **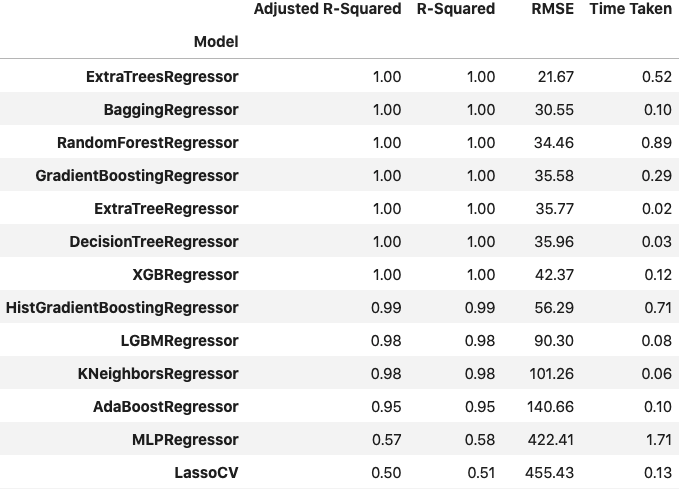 a)** |
| --- |
| **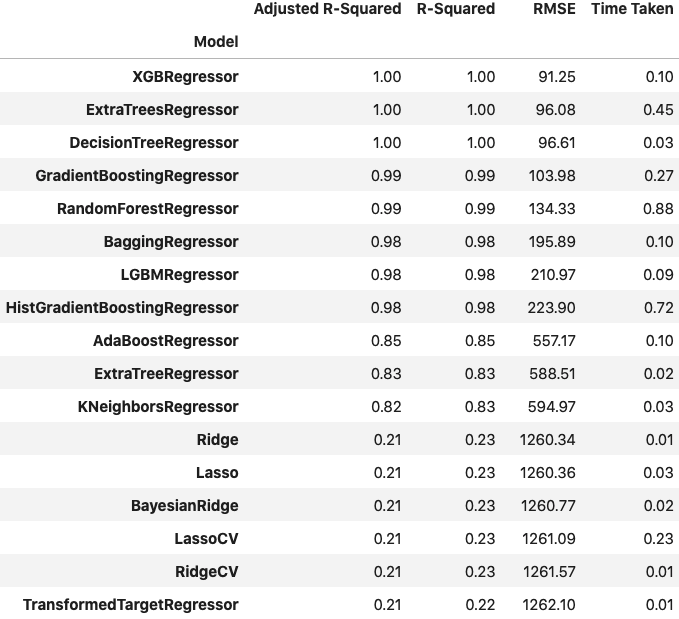 b)** |
| **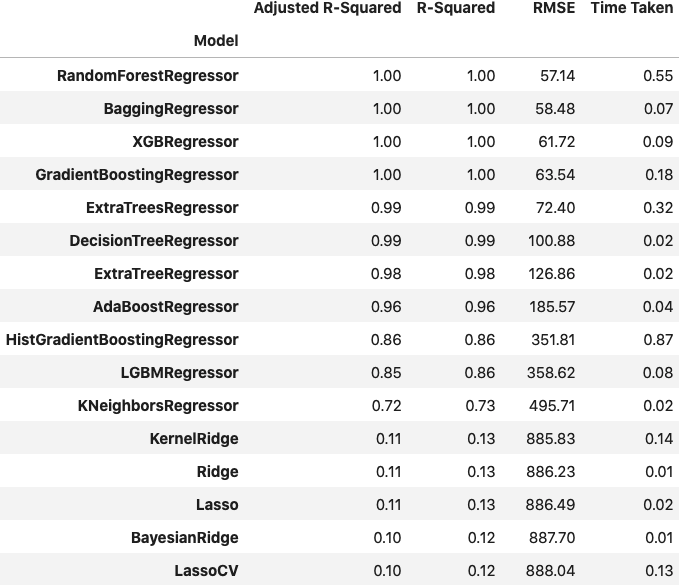 c)** |

**Supplementary Figure 4:** Summary of the best performing regression models obtained from Lazy Predict for three exemplary datasets. **a:** Tested for the ‘low pH’ process (Bioreactor 1). **b:** Tested for the pre-programmed pH control strategy 2 (Bioreactor 1). **c:** Tested for the autonomous logic pH strategy 2.

| **a)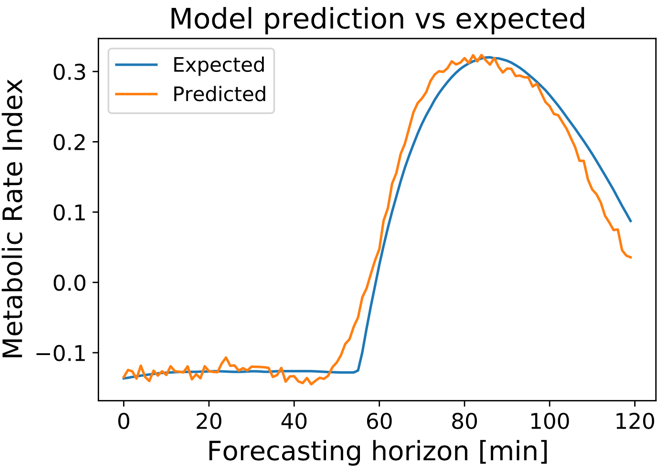** | **b)**  **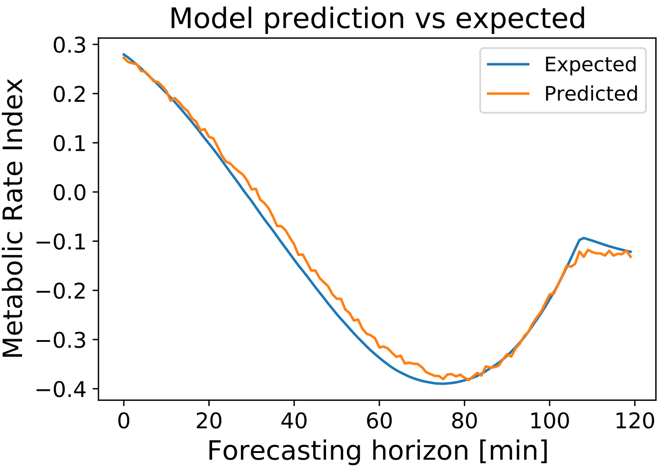** |
| --- | --- |
| **c)**  **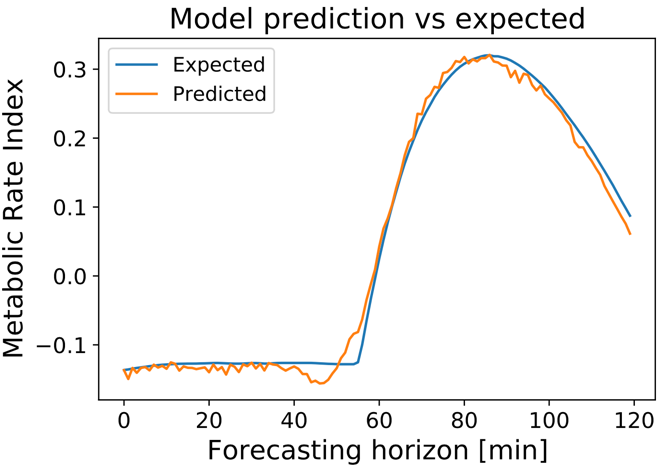** | **d)**  **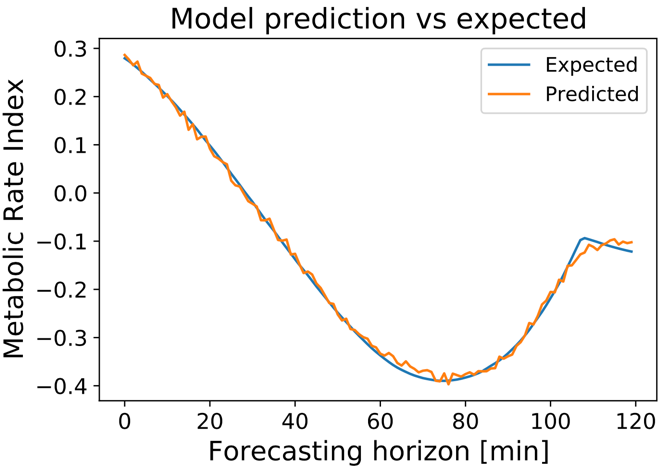** |

**Supplementary Figure 5:** Examples of MRI forecasting with LSTM for two different intervals of the ‘unoptimised’ process run in the first of two replicate bioreactors. **a, b:** Trained on past 24 hours. RMSE **a:** 0.027. RMSE **b:** 0.021. **c, d:** Trained on past 5 hours. RMSE **c:** 0.015. RMSE **d:** 0.011.

| **a)**  **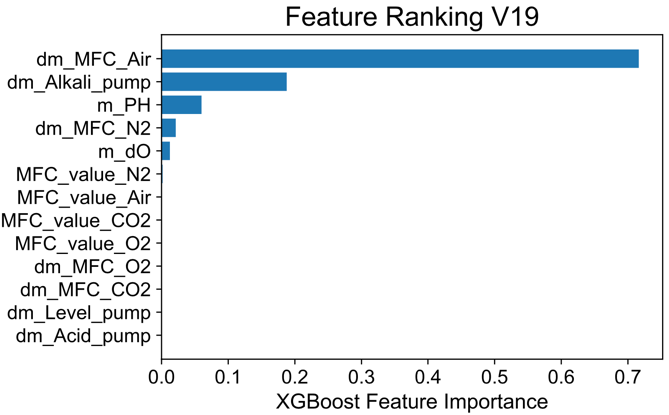** | **b)**  **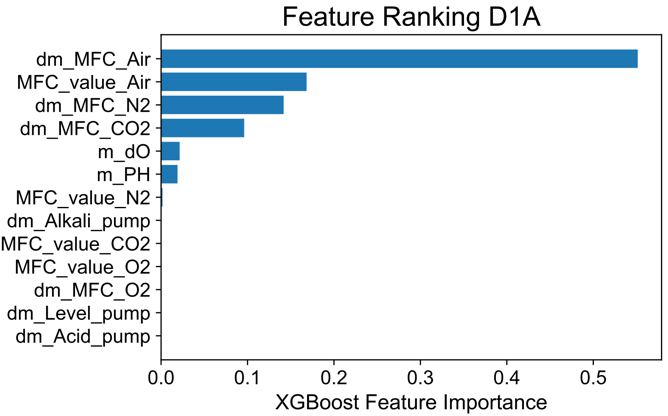** |
| --- | --- |
| **c)**  **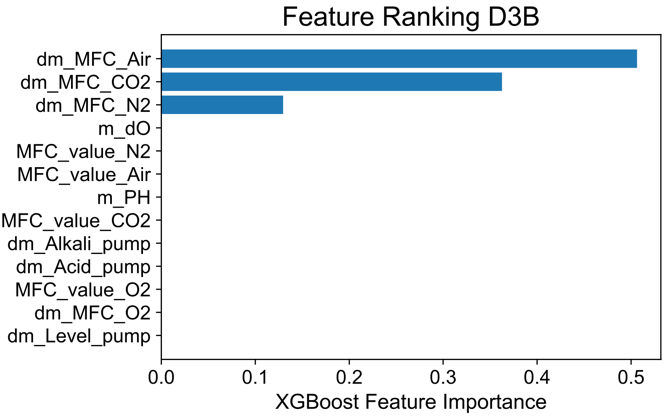** | **d)**  **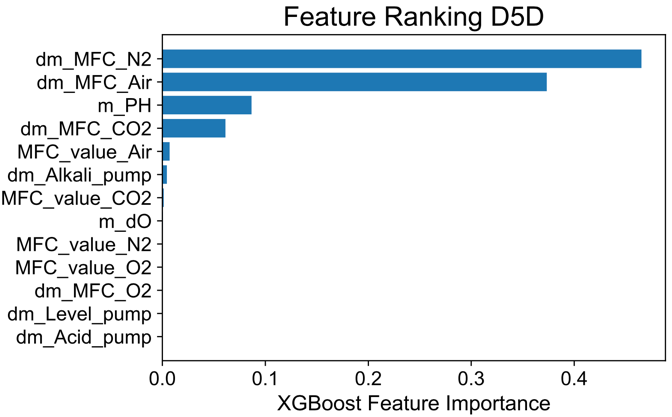** |

**Supplementary Figure 6:** XGBoost built-in feature importance scores of four regression models trained on different datasets. Multivariate regression models were implemented for the prediction of MRI.

| **a)**  **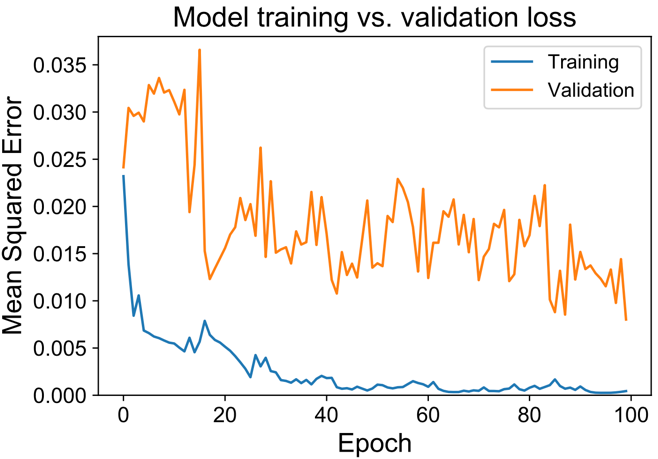** | **b)**  **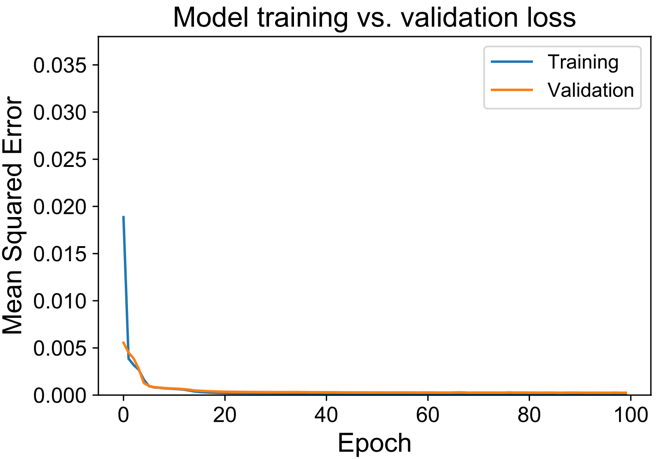** |
| --- | --- |
| **c)**  **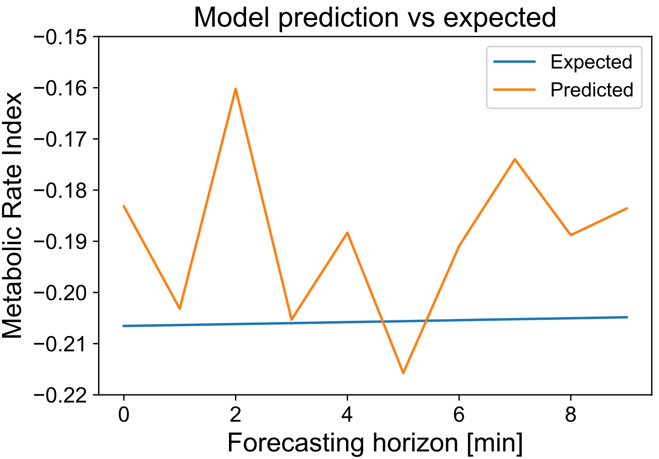** | **d)**  **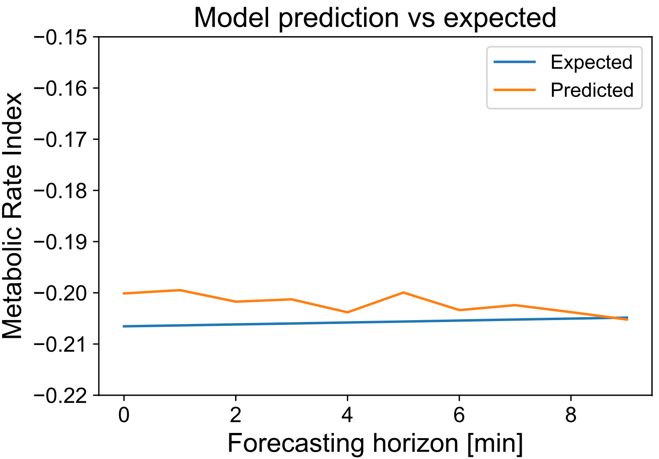** |
| **e)**  **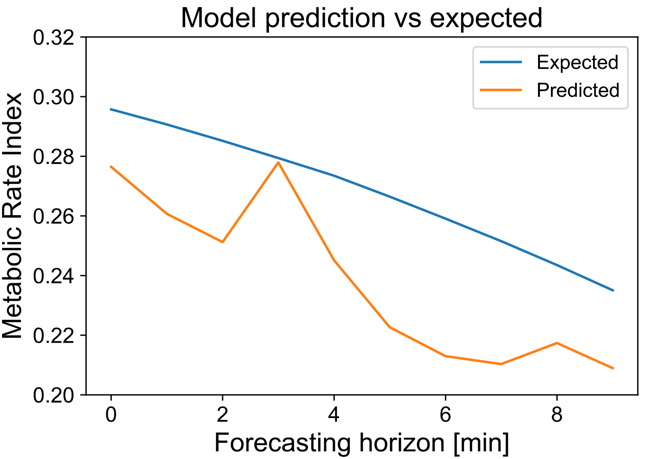** | **f)**  **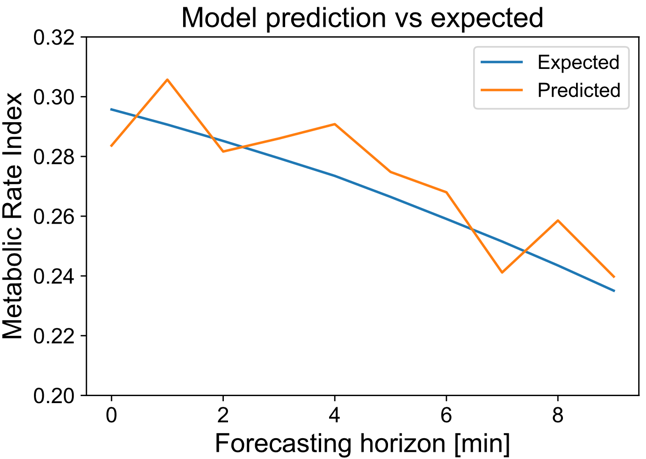** |

**Supplementary Figure 7:** Training and validation loss indicated by MSE for **a:** ordered and **b:** randomised model training on the ‘unoptimised’ process. Forecasting performance of the two different models for one test example, **c:** in-order trained model, RMSE: 0.022; **d:** randomised trained model, RMSE: 0.004. Forecasting performance of the two different models for another test example. **e:** in-order trained model, RMSE: 0.032; **f:** randomised trained model, RMSE: 0.011.
